# Discovery of annual growth in a modern olive branch based on carbon isotopes and implications for the Bronze Age volcanic eruption of Santorini

**DOI:** 10.1038/s41598-020-79024-4

**Published:** 2021-01-12

**Authors:** Yael Ehrlich, Lior Regev, Elisabetta Boaretto

**Affiliations:** 1 D-REAMS Radiocarbon Laboratory, Scientific Archaeology Unit, Weizmann Institute of Science, 7610001 Rehovot, Israel; 2 Max Planck-Weizmann Center for Integrative Archaeology and Anthropology, Weizmann Institute of Science, 7610001 Rehovot, Israel

**Keywords:** Plant sciences, Biogeochemistry

## Abstract

The volcanic eruption of Santorini in the Bronze Age left detectable debris across the Mediterranean, serving as an anchor in time for the region, synchronizing chronologies of different sites. However, dating the eruption has been elusive for decades, as radiocarbon indicates a date about a century earlier than archaeological chronologies. The identification of annual rings by CT in a charred olive branch, buried alive beneath the tephra on Santorini, was key in radiocarbon dating the eruption. Here, we detect a verified annual growth in a modern olive branch for the first time, using stable isotope analysis and high-resolution radiocarbon dating, identifying down to the growing season in some years. The verified growth is largely visible by CT, both in the branch’s fresh and charred forms. Although these results support the validity of the Santorini branch date, we observed some chronological anomalies in modern olive and simulated possible date range scenarios of the volcanic eruption of Santorini, given these observed phenomena. The results offer a way to reconcile this long-standing debate towards a mid-sixteenth century BCE date.

## Introduction

The volcanic eruption of Santorini in the Bronze Age was one of the largest in historical time^1,2^, spewing volcanic ash and sending pumice across the Mediterranean^3^. This debris found in stratified layers in archaeological sites serves as an absolute anchor in time, deeming layers beneath as “pre-eruption” and layers above as “post-eruption,” along with the artifacts they contain. According to the archaeological evidence synchronizing the Aegean with Egypt, the eruption should have occurred after the beginning of the New Kingdom in Egypt^4^. Based on extensive radiocarbon dating of dynastic Egypt, this date should fall between 1570–1544 BCE^5^.

However, radiocarbon dating places the eruption in the late seventeenth century BCE^6,7^, nearly a century in disagreement, generating a long-standing debate^8^ which has not been settled to this day^9–12^. The radiocarbon date is based on multiple samples of short-lived organic material from the destruction layer at Akrotiri^6^, as well as an olive branch, buried alive by tephra from the eruption^13^. The weighted average of the radiocarbon dates from 25 short-lived samples from Akrotiri (3345 ± 8)^6^ calibrated using the updated IntCal20 calibration curve^14^ provides a date range of 1630–1548 BCE (at 68.2% probability) or 1681–1542 BCE (at 95.4% probability). This date range spans more than a century, and thus is not very helpful in settling the debate.

The significance of wood buried alive as a sample for radiocarbon dating, as opposed to short-lived organic material such as an olive pit, is that wood is built over a number of years, providing a sequence in time rather than just one point. Matching multiple radiocarbon measurements to the radiocarbon calibration curve (termed “wiggle matching”^15^) generally gives a narrower date range compared to one radiocarbon measurement, which could potentially be matched to several areas on the calibration curve, resulting in a broader possible date range. The radiocarbon date for the eruption based on the olive branch, considering only that the samples taken from the branch are a sequence, is 1625–1567 BCE, at 1σ (68.2%) confidence interval^7^. This result is obtained by calibrating the radiocarbon age against the updated standard calibration curve, IntCal20^14^. This date range just barely includes the earliest possible date for the beginning of the New Kingdom.

Tree rings can store environmental data at an annual resolution and are thus an invaluable source for environmental as well as chronological data^16^. However, many trees do not form distinguishable annual growth rings, and the olive tree (*Olea europaea*) is thought to be among them^17^. This is most unfortunate as the olive is a long-lived fruit tree, one of the most common and characteristic species of the Mediterranean climate^18–20^, and the key piece of evidence for dating the Bronze Age eruption of Santorini. The debate surrounding the dating of the eruption sparked a renewed interest in determining whether or not olive trees produce annual growth rings^17^, as the first work published on the olive branch from Santorini relied on X-ray tomography for ring count, and subsequent modelling of radiocarbon dates^13^. It has previously been shown that subjective visual identification of olive growth rings is unreliable^17,21^.

Previous studies have shown an intra-annual fluctuation pattern of δ^13^C for numerous tree species^22–27^. These fluctuations are thought to be linked not only to δ^13^C fluctuations of atmospheric CO_2_ but also to changing environmental conditions, such as moisture, light, and temperature, which in turn affect the ratio of the partial pressure of CO_2_ in the intercellular spaces and that of atmospheric CO_2_, leading to a fractionation effect between the stable carbon isotopes. In addition, the exploitation of δ^13^C enriched energy reserves, such as starch during times of decreased photosynthetic activity, can lead to intra-annual variations in δ^13^C^24,28,29^.

We take advantage of this phenomenon and combine it with detailed structural studies of olive wood using micro-CT and chronological studies at a resolution of 1–2 years using radiocarbon dating. Dating at such high resolution is possible for a unique period of time beginning in the early 1960s, due to massive nuclear weapon testing in the atmosphere. This testing resulted in a sharp and nearly twofold increase in atmospheric radiocarbon levels, termed the “bomb peak”. This was followed by a gradual decrease, as atmospheric nuclear tests were banned^30^. Only in recent years has the atmospheric radiocarbon level approached “pre-bomb” levels. As the change in radiocarbon levels was relatively fast and dramatic, the difference between one year and another during the “bomb peak” is significant, which allows for a sequence of samples from this specific period to be radiocarbon dated at a nearly-annual (but sometimes sub-annual) resolution^30^.

## Results

### High-resolution radiocarbon dating of modern olive wood

**Figure 1:**
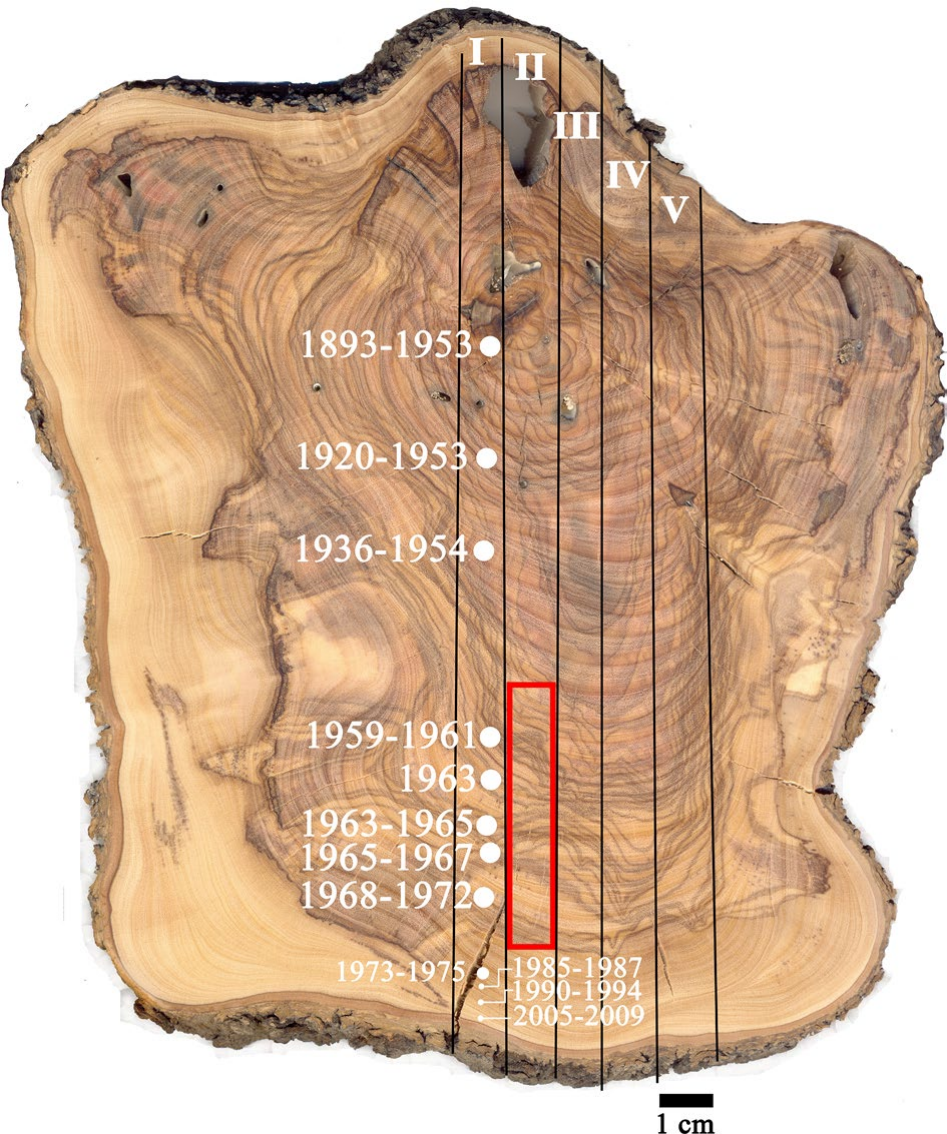
Olive branch cross-section. Five segments were cut along the section (I-V). Segment I was sampled at numerous points to obtain chronological data by radiocarbon dating. From segment II, 5 mm into the surface shown in the image, a 6 × 1x0.5 cm section (in red frame) was cut between the points dated 1959–1961 and 1968–1972, in order to contain the maximum point of the “bomb peak” period of increased atmospheric radiocarbon. This section from segment II was cut into a series of 96 independent samples that underwent radiocarbon dating and δ^13^C measurement. The tangentially parallel section was cut from above this segment, at the surface level shown in the image, for CT scanning following charring. The parallel section in segment III was CT scanned as fresh material. Segment IV was sampled for SEM–EDS analysis. A higher-quality image of the section is available in the supplementary material.

Numerous radiocarbon dates were obtained from a cross-section of a modern olive wood branch (Fig. 1), which was growing in northern Israel. Based on these dates, a 6 cm long segment was selected which contained the “bomb peak” years between 1957–1972 (red frame in Fig. 1). This segment was then cut with a microtome and divided into 96 contiguous samples, collected as pools of 30 µm thick sections of wood. A parallel segment was used for high-resolution micro-CT imaging.

**Figure 2:**
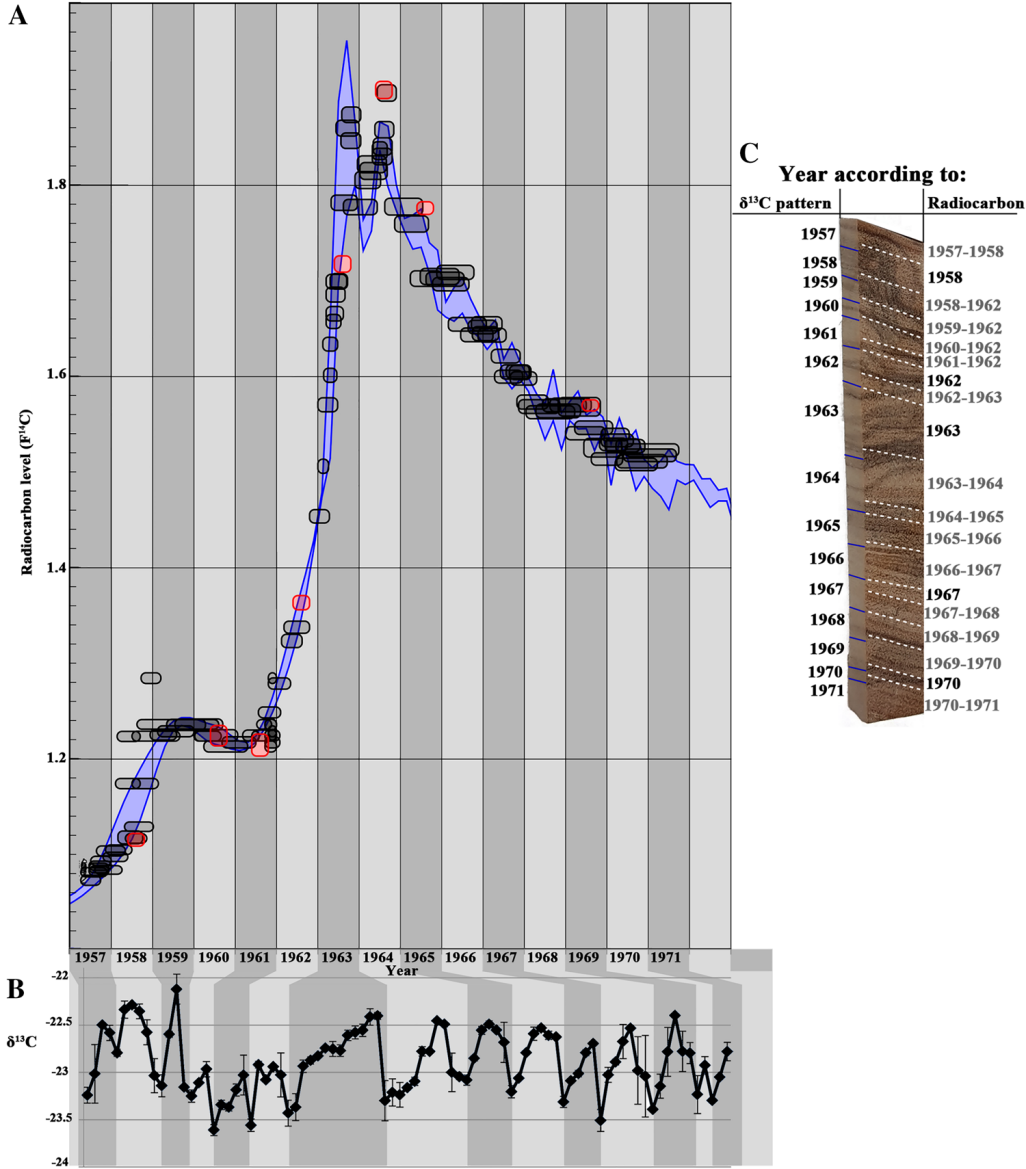
Radiocarbon and stable carbon isotopes data from olive wood. (**A**) A consecutive series of thin sections was sampled through an olive wood radius (96 samples in total) ranging from 1957–1971, a time period in which the “bomb peak” of increased atmospheric ^14^C can be used as reference (wide purple line). Olive wood samples from which α-cellulose was extracted and radiocarbon dated are indicated in black. As a control, a number of α-cellulose samples extracted from the latewood of pine growing in the same field as the olive tree were also analyzed for ^14^C and are marked in red. Results have been compared to the ^14^C calibration curve using OxCal v 4.2^31^ with the Bomb 13 NH2 calibration curve. (**B**) δ^13^C values for α-cellulose of all olive wood samples dated. The demarcation between years (light gray to dark gray) was evaluated according to minimum δ^13^C values and is aligned to the ^14^C data. (**C**) Radiocarbon calibrated years (modeled by OxCal 4.2) between dashed white lines and years according to δ^13^C variation pattern between blue markers.

Following α-cellulose extraction of all contiguous samples, aliquots were taken for ^14^C measurement and for δ^13^C composition determination. Radiocarbon results, presented as percent modern carbon (pMC), were calibrated and modeled with OxCal^31^. The pMC values obtained followed the radiocarbon “bomb peak” calibration curve closely and in a continuous manner (Fig. 2A), indicating consistent annual growth of the tree. A nearby growing pine (*Pinus halepensis*), a species known to have visibly distinguishable and reliable annual rings, was used as a reference. The pine ring widths were measured and cross-dated with a pine regional master chronology^32^ using standard dendrochronological methods. These dates were further verified by radiocarbon, measured from eight selected latewood α-cellulose samples (Fig. 2A), confirming the bomb peak curve shape and ruling out any considerable time lag in this region.

### Stable carbon isotopes ratio (δ^13^C) reveals sub-annual signal

Most significantly, δ^13^C values of the contiguous cellulose samples from the olive wood segment revealed a recurring cyclic pattern (Fig. 2B). Radiocarbon dates support the assignment of each minimum point of δ^13^C values to the border between growth years (Fig. 2B,C). This shows that indeed, olive trees do produce new wood annually and that this wood can be identified by the δ^13^C pattern, making this 15-year segment the first sequence of verified annual growth rings in olive wood. It should also be noted that it is anchored in time, thanks to the pine rings, and is not in a significant lag relative to the “bomb peak”.

### Seasonal cambial activity

**Figure 3:**
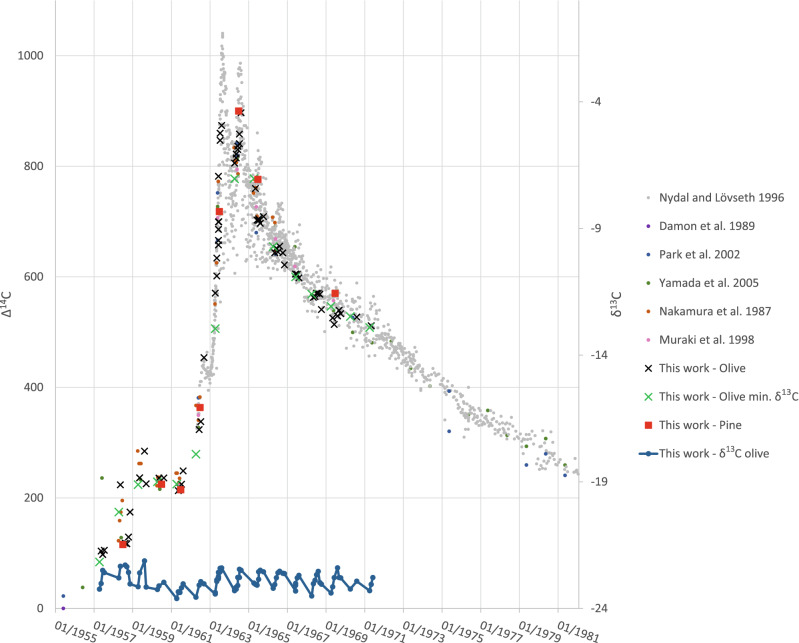
Radiocarbon data from olive wood compared to “bomb peak” calibration curve raw data. Radiocarbon levels for all olive wood contiguous samples are shown in comparison to all “bomb peak” raw data sources, which include atmospheric measurements^33^ and tree ring data^34–38^. Green data points correspond to minimum δ^13^C values (from Fig. 2), and represent the beginning of a growing season.

Comparing the radiocarbon data from the olive wood with the raw data of the calibration curve (as described in^30^ for northern hemisphere zone 2 which is based on^33–38^) enables an even higher resolution placement in time. We posit that the minimum points of δ^13^C values (Fig. 2B) indicate the beginning of the growing season. The corresponding radiocarbon values for all minimum δ^13^C values are marked as green in Fig. 3, such that each green data point represents the start of a new growing season. Arbitrarily setting these data points to a certain month within the previously established year, based on radiocarbon dating, would move all data points either left (earlier) or right (later) compared to the “bomb peak”. The best fit we found for our data and the “bomb peak” calibration curve was when we arbitrarily assigned these minimum δ^13^C points to April of the corresponding year. This is consistent with previous descriptions of the beginning of annual cambial activity in *olea europaea*^39,40^. In addition, there seems to be a cessation of growth during the end of the year until new growth is restarted in the following April. This is especially noticeable in 1963 and 1964, where if growth were continuous, we would expect to see a gradual decrease in radiocarbon levels, following the highest point (which also happens to be the latest in the year) of both peaks. Instead, there is a noticeable break in autumn (around September) until the following year begins.

### Identification of annual growth using CT scans of fresh and charred wood

As rings were originally identified in the olive branch found in Santorini^41^, the olive wood sections from segments II and III were scanned by micro-CT in order to observe changes in wood density. As shown in Fig. 3, the identifiable rings in the image generated by the micro-CT are easier to distinguish than those in the raw image. The banding in the raw image is, in part, due to dark-colored phenols which diffuse through multiple rings but are invisible to the micro-CT. It should be noted that an attempt to identify annual rings based on elemental fluctuations^17^ was carried out using scanning electron microscopy coupled with energy-dispersive X-ray spectroscopy (SEM–EDS) but did not reveal any ring patterns (Figure S1).

**Figure 4:**
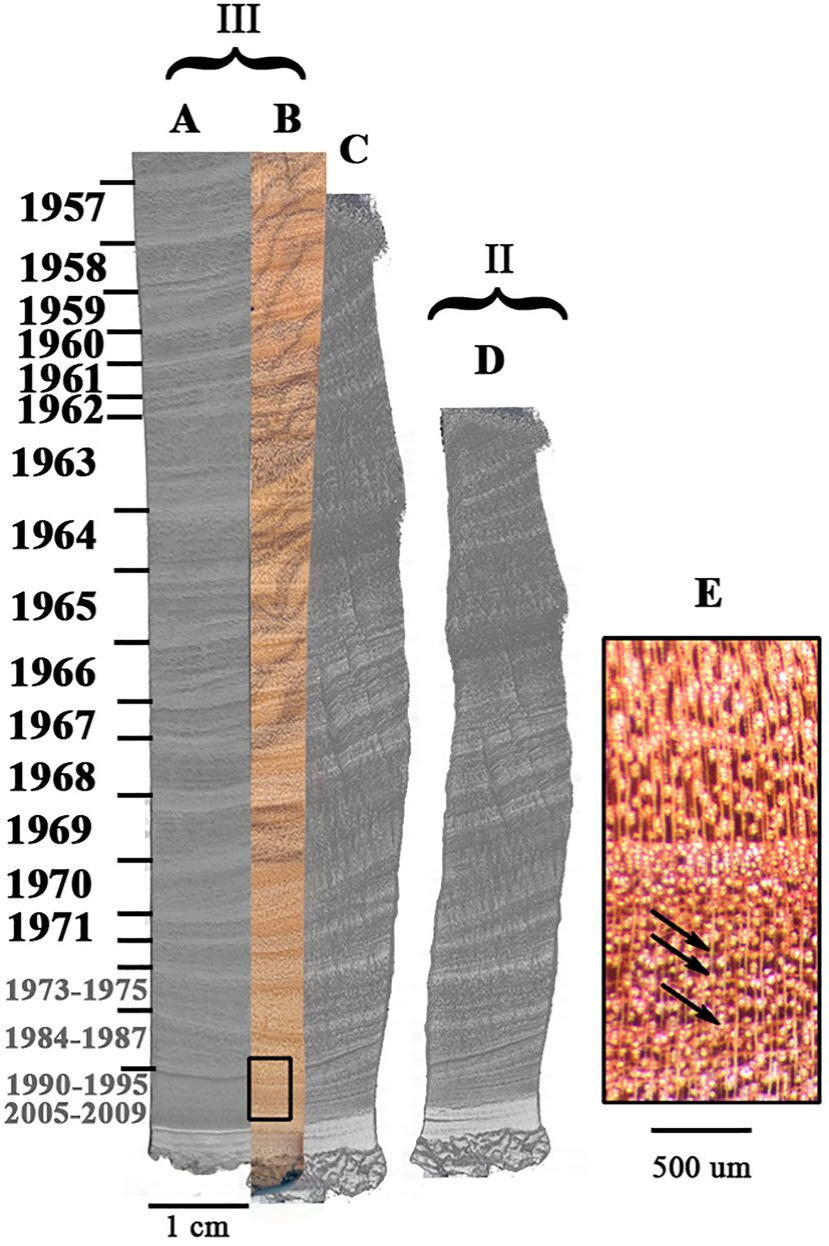
Micro-CT scan of olive wood. The micro-CT image of fresh wood (**A**) is shown in comparison to the actual image (**B**), both from segment III. (**D**) Charred sample from segment II after 30 min at 500 °C in continuous nitrogen flow. As charring caused warping and a decrease in size, (**C**) is the result of digitally stretching the original image (**D**), to more easily correlate the ring sequence to (**A**) and (**B**). Note that each marked ring is defined by a clear change in density, appearing as a strip of a lighter shade. Combining the radiocarbon data and δ^13^C patterns across the section, with the putative ring boundaries as seen in the micro-CT scan, each such defined ring was assigned a specific year. Year ranges in gray represent the calibration results from radiocarbon dating the cellulose extracted from sawdust encompassing the entire corresponding wood area for each result. (**E**) The magnified area outlined in (**B**), indicating examples of parenchyma bands, which can be interpreted as annual (see text). Ring widths between charred and fresh wood are compared in Figure S5 and Table S3.

With the data from radiocarbon and the δ^13^C pattern, we were able to identify the locations on the micro-CT image of transitions between years. It should be noted that ring boundaries are found at the point of an increase in density, observed as white streaks. Intra-annual density fluctuations (IADFs) are observed as narrow white streaks between the thicker areas of increased density which were identified as rings. The IADFs may be distinguished from changes of density, which do reflect a ring boundary, as a true ring boundary is thicker and more continuous, compared to the IADFs, which are thinner and, in some cases, discontinuous even across the 1 cm wide section analyzed (Fig. 4). We, therefore, show that annually deposited wood increments may generally be detected using micro-CT.

The olive branch (Fig. 1) was cut during 2013. Thus, the last ring in the section should be dated to this year. Indeed, the radiocarbon dating of cellulose closest to the bark was dated to 2009 (the latest date possible in the NH2 calibration curve^30^). However, in both the micro-CT image and the raw image, there are less than ten possible rings visible between the ring dated to 1971 and the outermost wood dated to 2005–2009. Thus, just within the last 1.5 cm of the section, there is a discrepancy of about 30 years between the number of rings identified by micro-CT and the years measured by radiocarbon (four bottom-most dates in Figs. 1 and 4). Under a stereomicroscope, 17 parenchyma bands were identified in the last 0.5 cm (black rectangle in Fig. 4B, enlarged in Fig. 4E, parenchyma bands marked by arrows). Parenchyma bands have previously been used as a marker of growth ring boundaries^42^. The first half of this section (Fig. 4E) was radiocarbon dated as bulk to between 1990–1995, and the second half was dated in the same manner to 2005–2009, corresponding roughly to the number of parenchyma bands identified. However, no parenchyma bands were detected in the preceding 1 cm (the area just above the black rectangle in Fig. 4A), dated to 1973–1975, or in fact, anywhere else on the radius. Thus, for this last region of 1.5 cm near the bark, we were not able to align radiocarbon years to physically identified rings in the olive wood.

We have furthermore found that the number of bands visible using micro-CT is not altered by charring, although the entire segment underwent non-homogenous shrinkage (Fig. 4D). It was not possible to align images of the charred and fresh segments by simply enlarging the image of the charred sample. Rather, different areas in the image of the charred segment required different enlargements (Fig. 4C) in order to correlate to the original, non-charred segment (Fig. 4A). The δ^13^C recurring annual pattern (Fig. 2B), which corresponds with the years determined by radiocarbon, was found to be relatively preserved in the charred segment (Fig. 4D), as the number of rings expected from the combination of radiocarbon and δ^13^C (Fig. 4C) is comparable to that identified by the δ^13^C pattern in the tangentially parallel segment (Fig. 4D). These results are illustrated in Figure S3, with the cellulose samples having a higher, more enriched average δ^13^C value of − 22.9‰, while the average δ^13^C value of the charred samples is − 24.0‰. The amplitude of δ^13^C fluctuation is generally higher in the cellulose samples, with an average standard deviation of 0.3‰, while the average standard deviation among the charred samples is 0.2‰.

### Simulation of dating scenarios based on modern olive wood chronological anomalies

The chronological anomalies we observed in modern olive wood included one CT-visible ring which, through radiocarbon dating, was discovered to hold about 20 years. In addition, previous work^43^ has shown that in a modern living olive branch, the outer wood, closest to the bark, can be off by a number of decades from the expected date. Thus, the wood, which should represent the last year of growth, may, in fact, be decades old, as olive wood can cease growth in one area of the circumference of a branch while continuing growth in another.

**Figure 5:**
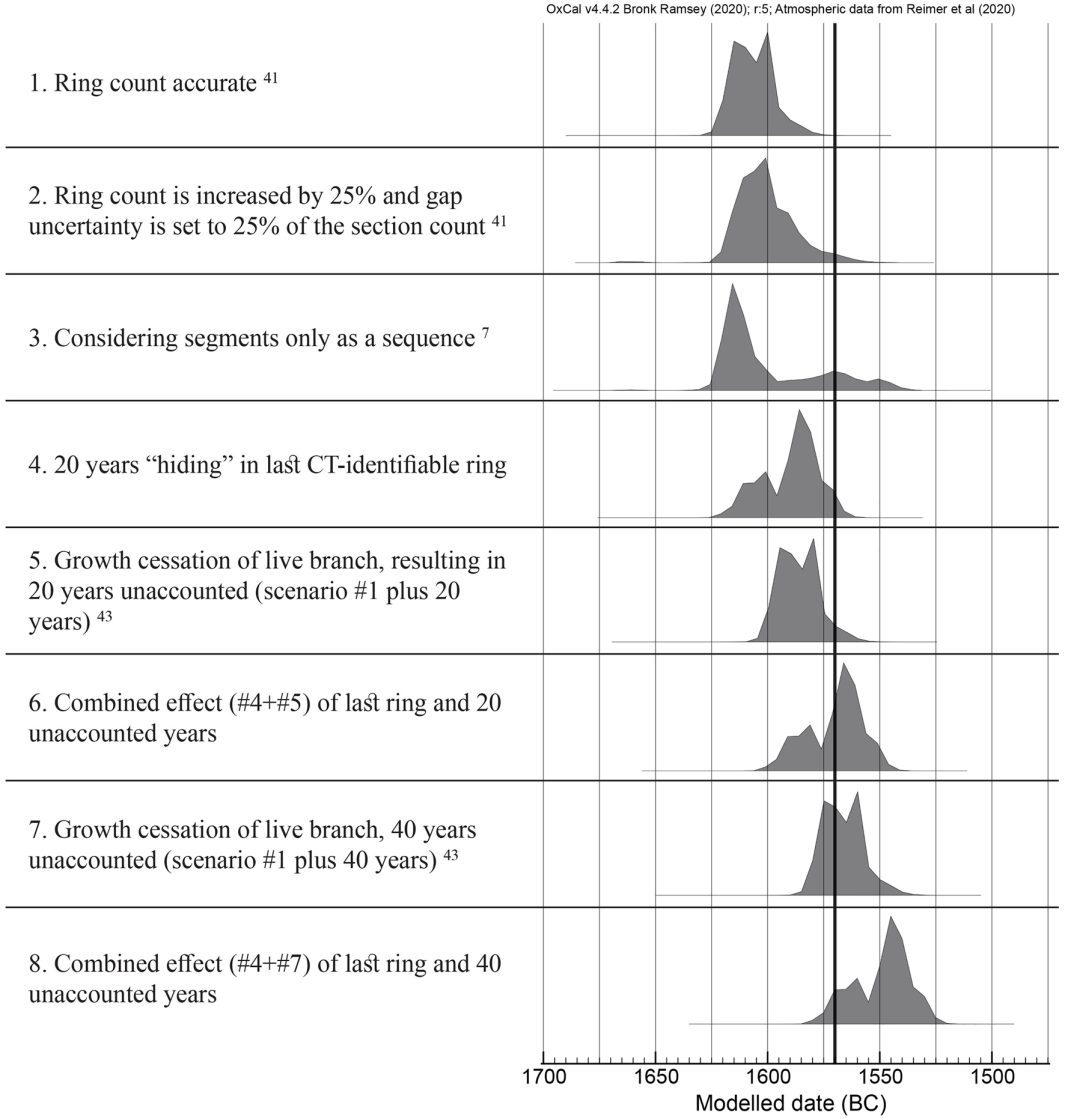
Modeling dating outcomes for the olive branch from Santorini. Given different scenarios based on chronological anomalies we observed in modern olive wood (scenarios 4–8), the radiocarbon date (uncalibrated age of 3331 ± 10) from the last segment of olive wood buried alive under the tephra at Santorini^13^ was calibrated and modeled. A bold line marks the date for the beginning of the New Kingdom at 1570 BCE.

As these scenarios were observed in modern olive wood, it is possible similar effects may have occurred in the olive branch found buried in tephra at Santorini, resulting in shifted date ranges that could include the beginning of New Kingdom more easily. We modeled the following scenarios (Fig. 5), first as was modeled by the original authors: 1. assuming ring count is accurate^13^; 2. increasing ring count by 25% and gap uncertainty to 25% of the section count^13^; 3. considering measured segments only as a chronological sequence, with no regard to ring count^7^. Next, we simulated the possible scenarios we observed in modern olive wood: 4. The last ring visible by CT actually includes 20 years; 5. branch ceased growth in the area sampled for radiocarbon, but continued growth elsewhere along the circumference of the branch for 20 years more^43^, until the eruption (adding 20 years to scenario 1); 6. a combined effect of the last ring comprising 20 years in addition to growth cessation 20 years previously; 7. Age discrepancy of 40 years around the circumference of the branch^43^; 8. The combined effect of the last ring comprising 20 years in addition to 40 years age discrepancy around the branch circumference.

**Figure 6:**
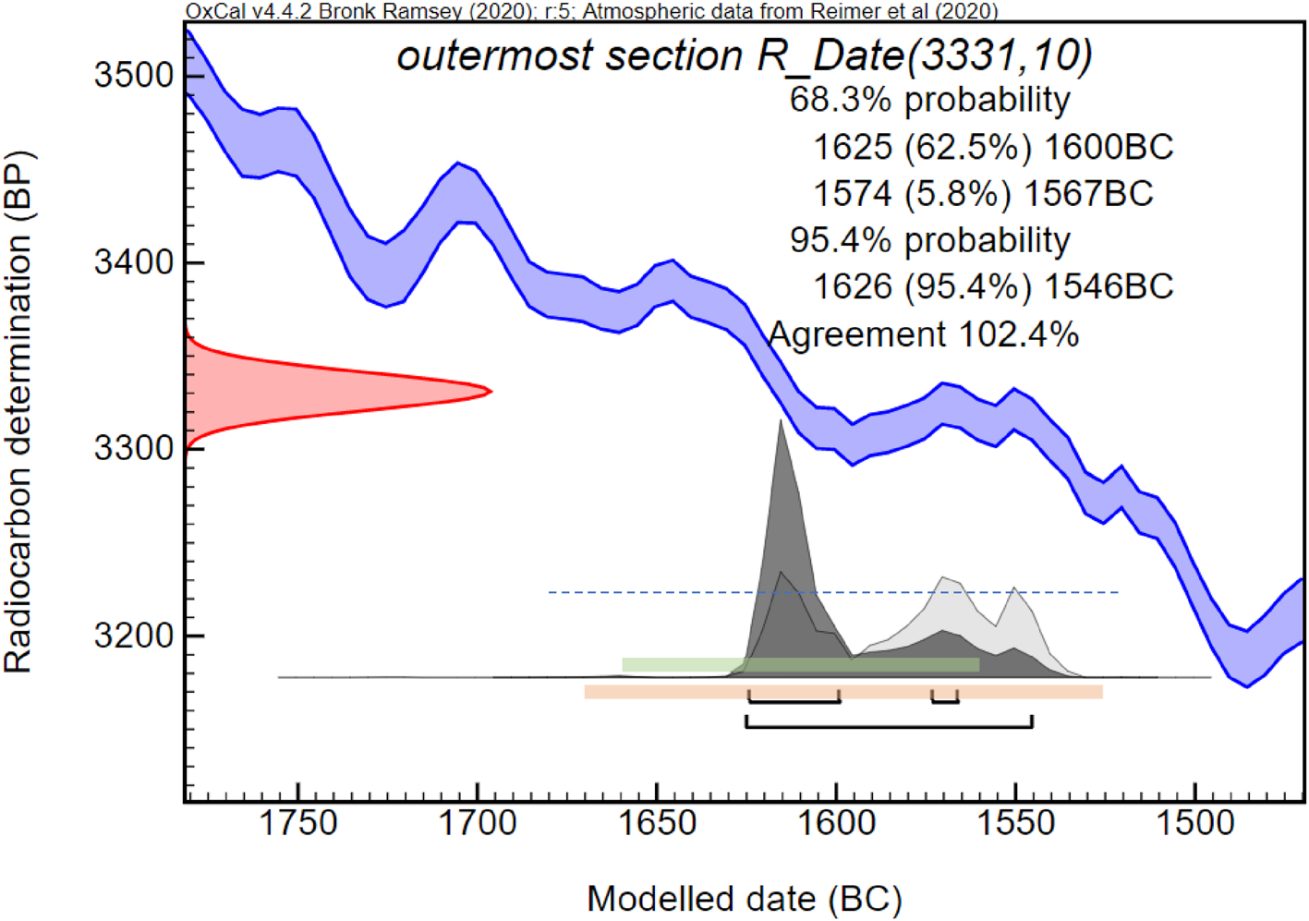
Probability distribution (dark gray) of modelled date range for the olive branch radiocarbon measurment^13^ (red) calibrated against IntCal20^14^. In the 1σ range, not all dates have an equal probability, as there are two peaks (1625–1600 with 62.5% out of the 68.2%; and 1574–1567 with only 5.8%). The 2σ range adds only one year to the beginning of the probable date range compared to 1σ, however, it adds another 21 years to the end of the range, which has a much lower probability than the pre-1600 range. Presenting the date range without specific probability information might be misleading. We therefore present date ranges which include probabilities of all included date ranges.

It should be noted that all dates within the range are not of equal probability. For example, the modeled and calibrated 2σ date range of scenario 3 (the only one in which the 1σ reaches a date range which can be settled with archaeological evidence-based chronology) using the recently published IntCal20^14^ is 1625–1567 BCE. It might then seem like the earliest possible date for the eruption at the beginning of the New Kingdom sometime after 1570 BCE could plausibly fit in this range. However, examining the probability distribution for this modeled date reveals a more complex picture (Fig. 6), due to the nature of the calibration curve, where not all dates in the date range are of equal probability.

## Discussion

Olive wood is frequently found as charred material in archaeological sites in the Mediterranean but is rarely exploited in dendrochronology due to the difficulty in objectively identifying annual rings. Here we utilized the “bomb peak” as an objective chronological reference for identifying annual rings in modern olive wood and unprecedentedly confirmed the annual fluctuating pattern of δ^13^C in fresh as well as charred wood. The intra-annual cyclicity of δ^13^C has been observed in many other tree species and is thus expected to be the result of a physiological mechanism, which can serve as a chronological tool independent of the “bomb peak”.

Based on the “bomb peak,” we have identified the growth season of olive wood, between April and September. It has been previously suggested that the olive would be out of sync with the northern hemisphere radiocarbon calibration curve, as it is harvested in autumn to winter^10^. However, as we have shown here, the growing season of the olive is springtime (during which olive pits form and harden^19^, making their time of harvest irrelevant), and therefore no shift from the regional calibration curve should be expected. This is indeed what we have observed – the contiguous radiocarbon measurements from olive wood match the “bomb peak” flawlessly.

The rings identified by micro-CT in this study were correlated with the rings confirmed by radiocarbon and the δ^13^C annual pattern. However, we have shown that nearly 30 years were masked in the last 1.5 cm of the wood radius. These were not detected by micro-CT or optical microscopy, but were evidenced by radiocarbon. Another example from a different olive tree growing nearby shows a similar problem of a deficit in the number of rings identified by micro-CT, compared with the number expected based on radiocarbon dating (Figure S2).

Considering it is possible for olive wood to contain a number of decades in the last CT-visible ring, as well as display an age discrepancy of a number of decades around the circumference of one living branch, these scenarios must be taken into account as possible explanations for the decades long unresolved disagreement between the radiocarbon-derived versus the archaeology-derived age of the Bronze Age eruption of Santorini. A crucial step forward was taken by Pearson et al.^44^, by publishing an annually resolved record of radiocarbon for the time period of 1700–1500 BCE. This work was combined in the updated IntCal20 standard radiocarbon calibration curve^14^. However, in most modeled scenarios, under a 1σ confidence level, the date range obtained from calibrating the last radiocarbon dated segment of the olive branch did not overlap with the date range of the beginning of the New Kingdom in Egypt.

Here, we modeled a number of scenarios based on the chronological anomalies we discovered in modern olive wood, and applied them to the olive branch radiocarbon results from Santorini. Calibrating the radiocarbon ages of the Santorini olive branch^13^ with the recently published annually resolved radiocarbon curve^44^ and IntCal 20^14^, and modeling the possible scenarios in which the measured date may not reflect the date of the eruption, results in date ranges which overlap with the date range suggested for the beginning of the New Kingdom in Egypt^5^.

This study shows that charred olive wood fragments, which are frequently found in archaeological sites around the Mediterranean, have the potential to reveal annual identifiable deposits. The δ^13^C pattern was relatively conserved through charring, as was found previously for almond^45^. In short sequences, the identification of a close estimate for ring count will enable carrying out “wiggle matching”^15^ for precise radiocarbon dating. Climatic information might also be extracted in longer sequences, either directly from the δ^13^C record or possibly from changes in cell structural properties^46^.

Correlation between δ^13^C and climate has been shown for many different species, but we have not been able to find a significant correlation between the δ^13^C values of the olive wood to climate data, such as the sum of annual precipitation or the average of maximum temperatures recorded for the area between the years 1957–1971 (Figure S4). In addition, we did not find a correlation between annual precipitation and widths of the corresponding growth rings during this period in the sampling location (Havat Hanania) in the olive or in the pine, a species often used in dendrochronology for this purpose (Figure S5). However, as a climate signal was not evident from the few pine trees sampled, the lack of correlation of olive tree rings in one branch to climate conditions should not be surprising, and should not discourage this direction of investigation.

In order to use the intra-annual δ^13^C fluctuation as an indication of the number of rings in olive wood, a more efficient method for high-resolution δ^13^C analysis of many continuous samples would be required, such as laser ablation-combustion-gas chromatography-isotope ratio mass spectroscopy (LA-C-GC-IRMS)^47^. In addition to the identification of olive wood growth season (spring through late summer), we have also noted a non-homogenous effect of charring on ring width size, which may have serious implications for dendrochronological studies of charred wood or paleoclimatic reconstructions based on ring width measurements carried out on other tree species. To our knowledge, the effect of charring on differential width change of tree rings has never been addressed.

In conclusion, by identifying annual and even seasonal growth in olive wood using high-resolution radiocarbon dating based on the “bomb peak”, we were able to provide plausible scenarios in which the radiocarbon dating of the olive branch found buried alive under the tephra of the Bronze Age volcanic eruption of Santorini can be reconciled with the archaeological evidence.

## Methods

### Experimental design

Our aim in this study was to find a method which may allow the detection of growth rings in olive wood, which would be confirmed as annual with radiocarbon dating. The highly resolved dating of a modern olive branch, besides serving as a reference for any potential annual ring detection method, also revealed some chronological anomalies. We then simulated these anomalies as possible scenarios for the olive branch found in Santorini, one of the most valuable remains for dating the Bronze Age eruption.

### Study area and sampling procedure

Olive (*Olea europeae*) and pine (*Pinus halepensis*) trees were sampled from Havat Hanania (N32°56.153′, E035°25.296′, 415 m) in northern Israel. The pine was cored at about 1.5 m above ground using a 5.15 mm increment borer. A branch of olive wood was cut transversally, into slices ~ 10 cm thick. Both pine and olive wood were polished to 1000 grit grade. The olive transverse wood section required a belt sander (Makita #9404) with five grades of grit (24, 40, 80, 150, 320) followed by polishing with a random orbit sander (Makita #BO5041) with four grades of grit (400, 600, 800 and 1000), while the pine required only the latter sander, beginning at 80 grit grade. A radial section of 10 × 650 × 5 mm sampled from the branch of one olive tree, which was cut down as part of landscaping work, as well as one pine core were cut into thin sections of 30 µm using a WSL-Lab Microtome^48^.

### Climate data

Precipitation and temperature information for Havat Hanania was retrieved from the Israeli Meteorological Service database. Precipitation data was obtained from weather stations 212,500 and 212,499 in Havat Hanania, with a few missing data points filled by using data from nearby stations 212,720 and 212,850. Daily temperature data was retrieved from the nearest weather station 4640, with missing data points taken from weather station 5360.

### α-cellulose extraction^43^

Between 20–40 mg of thin sections of wood were placed in 16 × 125 mm borosilicate glass test tubes. The tops of the tubes were stuffed with meshed glass wool. Acid–base-acid (ABA) pretreatment was carried out based on Southon and Magana (2010):samples were treated with aliquots of 5 ml 1 N HCl for 1 h, washed with DDW, followed by 5 ml of 0.1 N NaOH for 1 h, again washed with DDW and finally treated for 1 h with 5 ml 1 N HCl in a water bath at 70 °C to remove any carbon that may have adhered during the alkaline treatment. Immediately following the ABA pretreatment, hollocellulose was extracted using a modification to a variation of the Jayme-Wise method suggested by Southon and Magana (2010): 2.5 ml of 1 N HCl and 2.5 ml of 1 M NaClO_2_ were added to the samples which were then transferred to a water bath at 70 °C and left overnight. After bleaching, the samples were washed with DDW. The samples were treated with 6 ml of 5 N NaOH for 1 h, followed by washing with DDW and subsequently treated for 1 h with 5 ml of 1 N HCl in a water bath at 70 °C. The samples were then washed with DDW until reaching a neutral pH, and dried in an oven at 100 °C.

### Radiocarbon dating

Between 2–4 mg of α-cellulose were weighed into pre-baked (1 h at 900 °C) quartz tubes containing 200 mg CuO and oxidized to CO_2_ in a vacuum line at 900 °C for 3 h. CO_2_ pressure known to eventually result in ~ 1 mg carbon was transferred from each sample into tubes containing 1 mg of activated Co for graphitization. ^14^C content determination on the resulting graphite was carried out at the DANGOOR Research Accelerator Mass Spectrometry Laboratory at the Weizmann Institute^49^. All calculated ^14^C ages were corrected for isotopic fractionation based on the AMS stable carbon isotope ratio (δ^13^C value). Calibrated ages in calendar years were obtained from the IntCal20 (for pre-bomb) or Bomb 13 NH2 (for post-bomb) calibration curves^14,30^ using OxCal v 4.2^31^ or CALIBomb^30,50^.

### Stable isotope analysis

Cellulose samples of 0.3–0.55 mg were weighed into tin foil capsules (Elemental Microanalysis Ltd. 5 × 3.5 mm #D1015). Sample δ^l3^C values were determined with an elemental analyzer (Carlo Erba 1108) linked to a continuous flow isotope ratio mass spectrometer (Optima, Micromass, UK).

### X-ray computed tomography

Tree-ring visualization in olive wood was performed with a micro-CT scanner (CT-imaging, Germany). The protocol was performed with the following parameters:tubes voltage, 40 kV; tube current, 450 μA; exposure time, 90 ms.; effective voxel size, 0.080 mm. The charred segment was scanned with a micro-CT (Xradia Micro XCT-400) at an effective voxel size of 0.026 mm, where the parameters were:tubes voltage, 20 kV; tube current, 100 μA; exposure time, 20 s. The CT scans were performed at the In Vivo Imaging Center at the Weizmann Institute of Science. All images were viewed and analyzed with Avizo v. 9.2.

### Scanning electron microscopy

The sample was mounted on an aluminium stub with carbon tape, and carbon coated using an evaporator (Auto 306 Turbo, Edwards, United Kingdom). Samples were imaged with a Supra 55 FEG SEM (Zeiss) using a secondary electron detector (2–5 kV), in combination with an EDS detector (Oxford) for elemental analysis. These experiments were carried out at the Electron Microscopy Unit of the Weizmann Institute of Science.

## Supplementary information

Supplementary Information 1.
