# Supplementary material for: Discovery of annual growth in a modern olive branch based on carbon isotopes and implications for the Bronze Age volcanic eruption of Santorini
Source: Sci Rep. 2021 Jan 12;11:704. doi: 10.1038/s41598-020-79024-4 (PMC7804959; doi:10.1038/s41598-020-79024-4)
Supplement: Supplementary file 1 — Supplementary Information 1. [file 41598_2020_79024_MOESM1_ESM.docx]

Discovery of annual growth in a modern olive branch based on carbon isotopes and implications for the Bronze Age volcanic eruption of Santorini

**Yael Ehrlich, Lior Regev and Elisabetta Boaretto**

Supplementary Materials

**
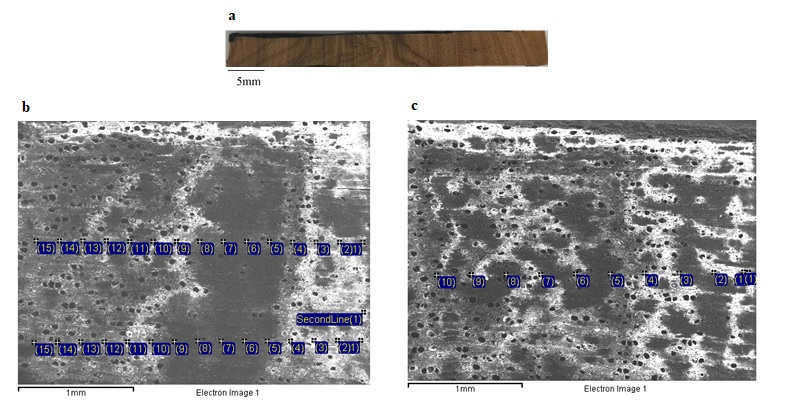
**

**B**

5mm


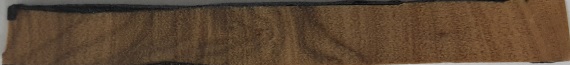


**Fig. S 1. SEM-EDS olive wood scan.** Scans of segment IV were obtained using a secondary electron detector. The section analyzed includes the “bomb peak” period. The scans show no observable spatial chemical signal along the radius. **(A)** image of the fresh segment of olive wood for SEM-EDS. **(B,C)** Representative SEM-EDS scans of sub-sections of (A), both near the middle of the section.

**
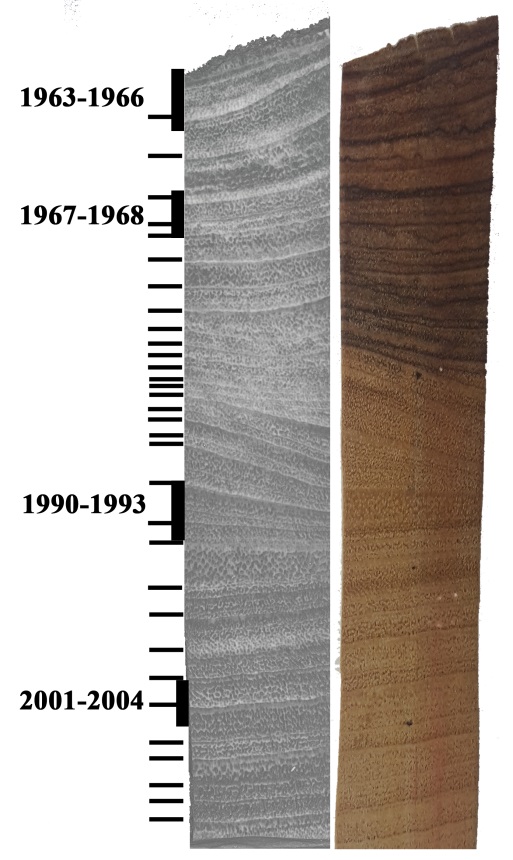
**

**Fig. S 2. Micro-CT scan of an additional olive tree.** This sample was collected from a tree at the same site as the tree analyzed in the main text. This branch cross section was radiocarbon dated at numerous points along the radius. The area from which material collected for dating of each point is indicated by the length of the vertical bars. Horizontal lines indicate the locations of observed fluctuations in density. Note that the number of rings identified between the points 1963-1966 and 1967-1968, match the number of years indicated by radiocarbon. Between the points 1990-1993 and 2001-2004 there is a deficit of at least one ring, to account for the distance in time between the points measured by radiocarbon. Between the points 1967-1968 and 1990-1993 there is a deficit of at least 10 rings which we were not able to identify with micro-CT.

**Fig. S 3. Comparison of δ^13^C values from cellulose and charred wood.** Left: δ^13^C values for cellulose (black) and charred wood (blue) from tangentially parallel olive wood segments. Right: from fig 3 in the main text. The unshaded area was previously analyzed for δ^13^C from cellulose (Fig. 2B) and here compared with charred material (D and E, CT scan and actual image, respectively), comprising the time period between 1957-1966. Note there are 10 clear peaks for cellulose (black) and 8 clear peaks for the charred parallel segment.


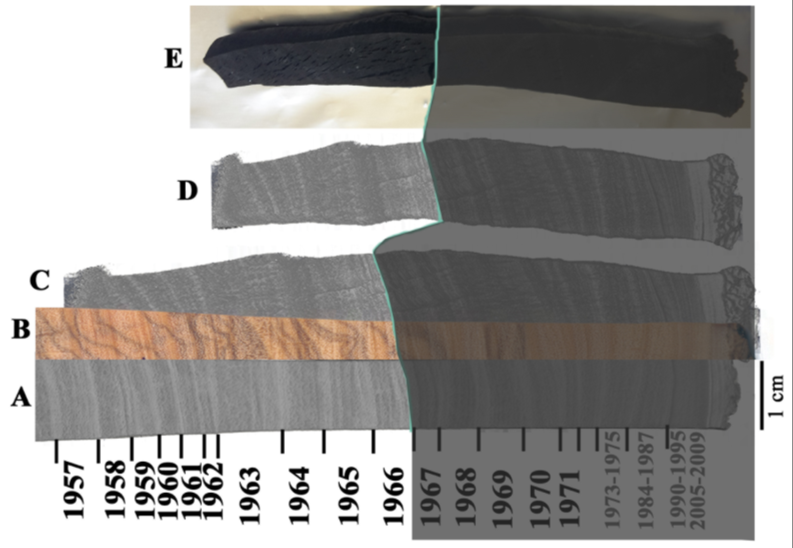

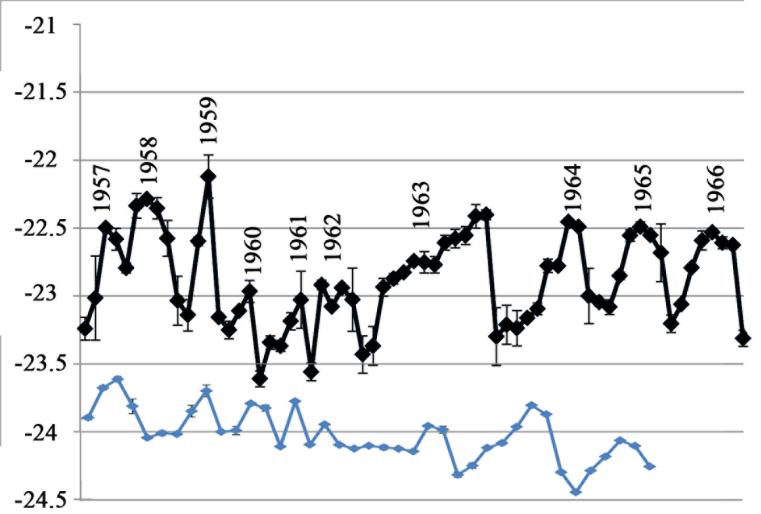


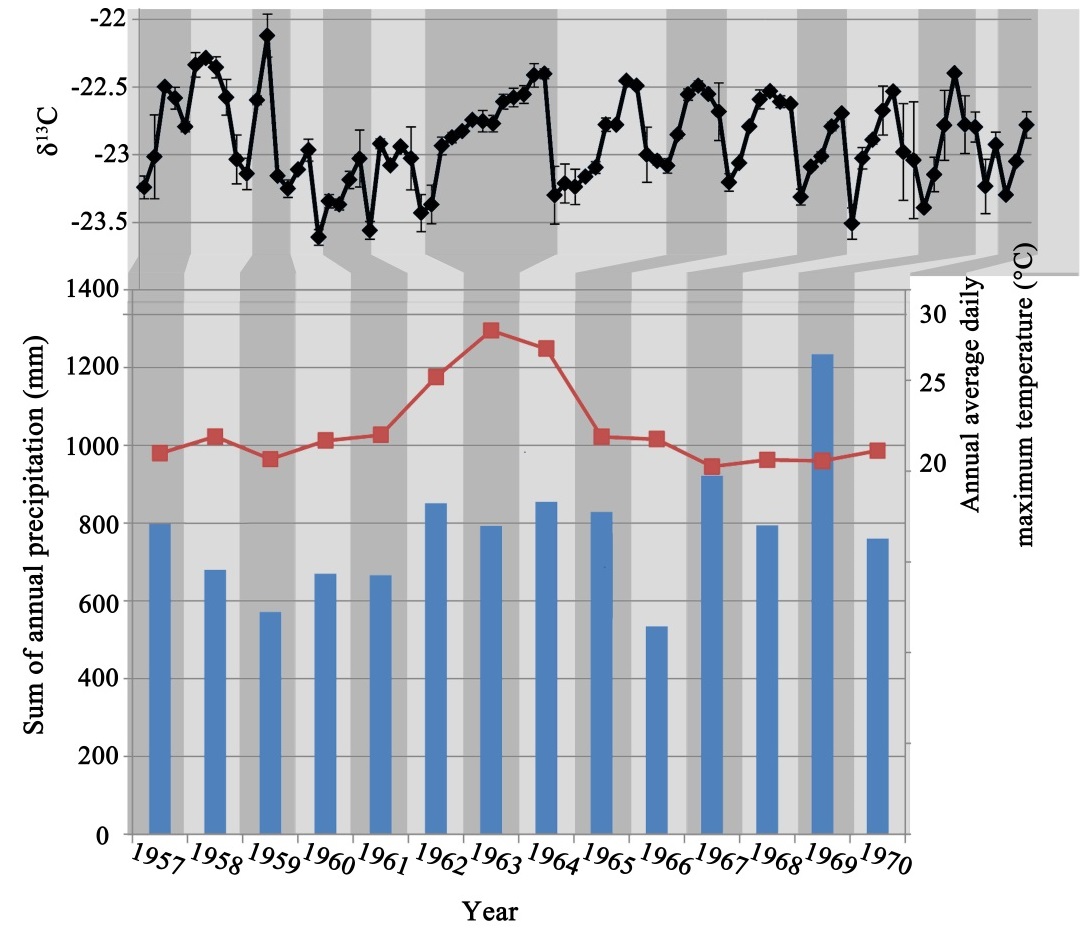


**Fig. S 4. Precipitation and temperature data for Havat Hanania, relative to δ^13^C pattern**. No correlation is observed between annual rainfall (blue bars) and δ^13^C, or between annual average daily maximum temperature (red squares) and δ^13^C.

**
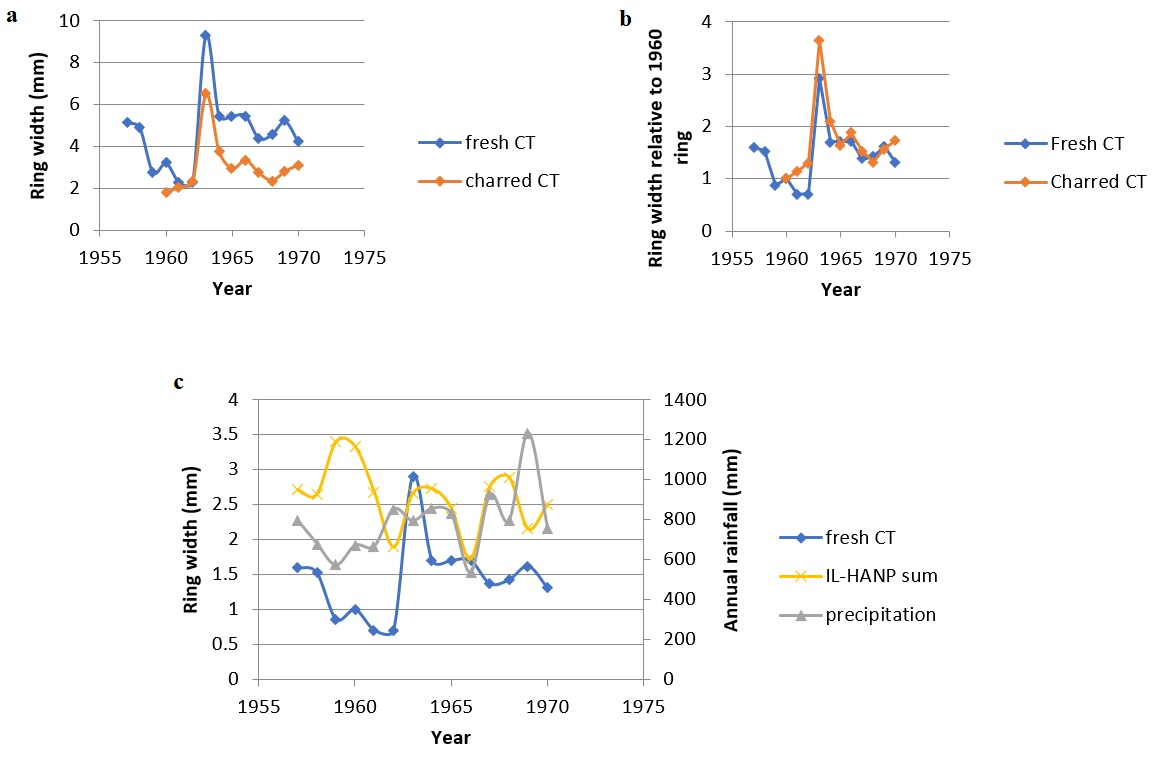
**

**Fig. S 5. Ring width correlation with precipitation.** (a) Absolute ring widths in mm, comparing the widths measured in the images acquired by CT scanning of fresh wood and the same wood sample after charring. (b) Samples as in A, but here with ring widths relative to the ring of year 1960, arbitrarily assigned with the value of 1. (c) Absolute ring width values of pine, relative values of olive, overlaid on precipitation data for Havat Hanania.

**C**

**A**

Tables S1-S6

**Table S1.** Percent modern carbon (pMC) values for samples of section I from HAN2B cross-section, calibrated using CALIBomb ^44^, and modelled with OxCal v 4.2 ^27^, as a chronological sequence, with no ring number information. All presented dates are cal AD.

| Lab no. | Putative ring no. | pmc | pmc +/- | cal 1σ | cal 2σ | OxCal modelling |
| --- | --- | --- | --- | --- | --- | --- |
| RT-7207 | 3 | 99.295 | 0.478 | [ 1697 : 1724]0.282  [ 1815 : 1835]0.201  [ 1877 : 1917]0.509  [ 1955 : 1955]0.009 | [ 1686 : 1731]0.254  [ 1808 : 1927]0.739  [ 1954 : 1956]0.008 | 1893-1953 |
| RT-7208 | 9 | 97.605 | 0.475 | [ 1660 : 1682]0.214  [ 1737 : 1759]0.179  [ 1761 : 1804]0.428  [ 1936 : 1954]0.179 | [ 1644 : 1697]0.244  [ 1724 : 1815]0.510  [ 1834 : 1878]0.054  [ 1916 : 1955]0.192 | 1920-1953 |
| RT-7209 | 18 | 97.348 | 0.471 | [ 1647 : 1680]0.391  [ 1740 : 1741]0.013  [ 1763 : 1801]0.442  [ 1938 : 1952]0.154 | [ 1528 : 1552]0.019  [ 1633 : 1694]0.332  [ 1727 : 1813]0.478  [ 1839 : 1841]0.002  [ 1854 : 1858]0.003  [ 1862 : 1866]0.003  [ 1918 : 1955]0.164 | 1936-1954 |
| RT-7210 | 30 | 122.984 | 0.352 | [ 1959.37 : 1961.15]0.597  [ 1961.51 : 1961.63]0.051  [ 1982.90 : 1983.86]0.352 | [ 1959.21 : 1961.69]0.577  [ 1982.17 : 1985.02]0.423 | 1959-1961 |
| RT-7615 | 33 | 155.235 | 0.515 | [ 1968.26 : 1968.51]0.120 [ 1968.79 : 1969.96]0.780 [ 1970.27 : 1970.40]0.099 | [ 1963.23 : 1963.30]0.025  [ 1967.03 : 1967.04]0.001  [ 1968.02 : 1969.98]0.866  [ 1970.23 : 1970.44]0.104  [ 1970.77 : 1970.80]0.004 | 1963 |
| RT-7616 | 35 | 178.818 | 0.507 | [ 1963.45 : 1963.49]0.037 [ 1963.90 : 1964.40]0.402 [ 1964.71 : 1965.04]0.453 [ 1965.41 : 1965.54]0.108 | [ 1963.43 : 1964.41]0.441  [ 1964.64 : 1965.58]0.546  [ 1965.82 : 1965.88]0.013 | 1963-1965 |
| RT-7617 | 37 | 164.545 | 0.716 | [ 1963.33 : 1963.36]0.027 [ 1966.09 : 1966.13]0.028 [ 1966.67 : 1967.36]0.871 [ 1967.58 : 1967.64]0.074 | [ 1963.31 : 1963.39]0.034 [ 1965.68 : 1967.66]0.966 | 1965-1967 |
| RT-7618 | 43 | 151.939 | 0.491 | [ 1963.20 : 1963.24]0.048 [ 1968.90 : 1968.99]0.051 [ 1969.65 : 1969.73]0.090 [ 1969.98 : 1970.89]0.534 [ 1971.35 : 1971.45]0.092 [ 1971.66 : 1971.76]0.086 [ 1971.96 : 1972.09]0.099 | [ 1963.16 : 1963.28]0.055 [ 1968.44 : 1968.45]0.001 [ 1968.87 : 1969.02]0.061 [ 1969.63 : 1969.75]0.073 [ 1969.97 : 1971.11]0.502 [ 1971.31 : 1972.22]0.308 | 1968-1972 |
| RT-7619 | 49 | 141.55 | 0.489 | [ 1962.75 : 1962.86]0.096 [ 1973.90 : 1974.82]0.853 [ 1975.48 : 1975.55]0.051 | [ 1962.68 : 1962.91]0.098 [ 1973.02 : 1975.12]0.791 [ 1975.37 : 1975.80]0.111 | 1973-1975 |
| RT-7283 | 50 | 119.553 | 0.367 | [ 1985.09 : 1987.72]1.000 | [ 1958.73 : 1959.28]0.089 [ 1984.11 : 1987.98]0.911 | 1985-1987 |
| RT-7211 | 50-58 | 113.844 | 0.63 | [ 1991.10 : 1993.78]1.000 | [ 1958.01 : 1958.78]0.067 [ 1990.34 : 1995.72]0.933 | 1990-1994 |
| RT-7284 | 58-67 | 106.013 | 0.36 | [ 1957.04 : 1957.25]0.066 [ 2005.26 : 2008.86]0.934 | [ 1956.82 : 1957.51]0.111 [ 2005.17 : 2009.52]0.889 | 2005-2009 |

**Table S2**. pMC of latewood samples from pine rings of a tree growing in Havat Hanania. Results were modelled in OxCal using D_sequence with known annual gaps, according to the annual rings, was inserted as additional input.

| Lab no. | Year as derived from dendrochronology | pMC | pMC ± 1σ | OxCal modelling (D_seq) | |
| --- | --- | --- | --- | --- | --- |
|  |  |  |  | from | to |
| RT 8222 | 1958 | 111.568 | 0.337 | 1958 | 1958 |
| RT 8223 | 1960 | 122.46 | 0.542 | 1960 | 1960 |
| RT 8224 | 1961 | 121.474 | 0.583 | 1961 | 1961 |
| RT 8225 | 1962 | 136.36 | 0.382 | 1962 | 1962 |
| RT 8226 | 1963 | 171.796 | 0.433 | 1963 | 1963 |
| RT 8227 | 1964 | 189.991 | 0.449 | 1964 | 1964 |
| RT 8228 | 1965 | 177.631 | 0.333 | 1965 | 1965 |
| RT 8231 | 1969 | 156.993 | 0.3 | 1969 | 1969 |

.

**Table S3.** **Ring width comparison as measured from CT scans of fresh and charred wood.** Three measurments were carried out for each ring, and averaged.

|  | Ring width (mm) | | | | | | | | | |
| --- | --- | --- | --- | --- | --- | --- | --- | --- | --- | --- |
|  | CT fresh | | | | | CT charred | | | | |
| Ring | Measurment 1 | Measurment 2 | Measurment 3 | average | SD | Measurment 1 | Measurment 2 | Measurment 3 | average | SD |
| 1957 | 4.92 | 5.29 | 5.19 | 5.13 | 0.15 |  |  |  |  |  |
| 1958 | 4.73 | 5.01 | 4.96 | 4.90 | 0.12 |  |  |  |  |  |
| 1959 | 2.82 | 2.89 | 2.60 | 2.77 | 0.12 |  |  |  |  |  |
| 1960 | 3.33 | 3.14 | 3.14 | 3.20 | 0.09 | 1.89 | 1.79 | 1.68 | 1.79 | 0.08 |
| 1961 | 2.29 | 2.28 | 2.20 | 2.26 | 0.04 | 2.01 | 2.02 | 2.11 | 2.05 | 0.05 |
| 1962 | 2.23 | 2.26 | 2.31 | 2.26 | 0.03 | 2.39 | 2.44 | 2.14 | 2.32 | 0.13 |
| 1963 | 9.29 | 9.27 | 9.37 | 9.31 | 0.04 | 6.56 | 6.21 | 6.72 | 6.49 | 0.21 |
| 1964 | 5.69 | 5.36 | 5.30 | 5.45 | 0.17 | 3.52 | 3.51 | 4.15 | 3.72 | 0.30 |
| 1965 | 5.32 | 5.57 | 5.47 | 5.45 | 0.10 | 3.07 | 2.95 | 2.80 | 2.94 | 0.11 |
| 1966 | 5.37 | 5.41 | 5.61 | 5.46 | 0.10 | 3.47 | 3.28 | 3.26 | 3.34 | 0.09 |
| 1967 | 4.16 | 4.54 | 4.48 | 4.39 | 0.17 | 2.83 | 2.56 | 2.78 | 2.72 | 0.12 |
| 1968 | 4.41 | 4.65 | 4.63 | 4.57 | 0.11 | 2.13 | 2.51 | 2.45 | 2.36 | 0.17 |
| 1969 | 5.22 | 5.40 | 4.95 | 5.19 | 0.18 | 2.65 | 2.86 | 2.91 | 2.81 | 0.11 |
| 1970 | 4.32 | 4.08 | 4.21 | 4.20 | 0.10 | 2.89 | 3.03 | 3.38 | 3.10 | 0.20 |

**Table S4.** Correlation analysis between annual precipitation data and ring widths of olive rings, as measured from CT scans, and pine ring widths, from Havat Hanania. No significant correlation between precipitation data and olive or pine ring widths for the years 1957-1970 was found.

|  | *fresh* | *charred* | *precipitation* | *pine* |
| --- | --- | --- | --- | --- |
| fresh | 1 |  |  |  |
| charred | 0.918019 | 1 |  |  |
| precipitation | 0.205597 | 0.022832 | 1 |  |
| pine | -0.1552 | -0.09667 | -0.25462 | 1 |

**Table S5.** Details of Israel Meterological Service weather stations, from which precipitation and temperature data were obtained.

| Station name | Station number | **N** | **E** | **Height (asl)** |
| --- | --- | --- | --- | --- |
| Hanania | 212499  212500 | 35.4205º | 32.9363º | 410 |
| Karmiel | 212720 | 35.3055º | 32.9124º | 210 |
| Maghar | 212850 | 35.4048º | 32.8898º | 275 |
| Tavor Kadoori | 5360 | 35.4069º | 32.7053º | 145 |
| Zefat Har kenaan | 4640 | 35.5070º | 32.9800º | 936 |

**Table S6.** All olive wood samples which were radiocarbon dated. Presented are the pMC values with their respective error range, unmodelled calibrated ages according to CALIBomb ^44^ and calibrated ages modelled with OxCal v 4.2 ^27^. All dates are Cal AD.

| **RT#** | **pMC** | **pMC+-** | **CALIBomb** | | **OxCal** | |
| --- | --- | --- | --- | --- | --- | --- |
|  |  |  | Cal 1σ | Cal 2σ | Cal 1σ | Cal 2σ |
| 8262 | 107.32 | 0.262 | [ 2002.94:2005.02] 1.000 | [ 1957.19:1957.78] 0.081 [ 2002.16:2002.42] 0.027 [ 2002.74:2005.95] 0.887 [ 2006.27:2006.45] 0.005 | 1957:1957 | 1957:1957 |
| 8263 | 108.163 | 0.241 | [ 2001.11:2001.45] 0.204 [ 2001.92:2003.04] 0.764 [ 2003.48:2003.55] 0.032 | [ 1957.45:1957.91] 0.066 [ 2001.05:2004.23] 0.934 | 1957:1957 | 1957:1957 |
| 8264 | 108.301 | 0.244 | [ 2001.09:2001.47] 0.274 [ 2001.89:2002.90] 0.726 | [ 1957.49:1957.93] 0.064 [ 2001.01:2004.20] 0.936 | 1957:1957 | 1957:1957 |
| 8265 | 108.604 | 0.254 | [ 2001.01:2002.17] 0.826 [ 2002.43:2002.74] 0.174 | [ 1957.51:1958.05] 0.071 [ 2000.16:2003.09] 0.922 [ 2003.35:2003.57] 0.007 | 1957:1957 | 1957:1957 |
| 8266 | 108.851 | 0.241 | [ 2000.19:2002.04] 1.000 | [ 1957.55:1958.03] 0.066 [ 1999.18:1999.39] 0.010 [ 2000.01:2002.89] 0.924 | 1957:1957 | 1957:1958 |
| 8267 | 109.358 | 0.239 | [ 1999.29:1999.40] 0.049 [ 1999.91:2001.05] 0.924 [ 2001.51:2001.57] 0.027 | [ 1957.59:1958.12] 0.064 [ 1998.31:1998.39] 0.007 [ 1998.85:2001.91] 0.930 | 1957:1957 | 1957:1958 |
| 8269 | 108.407 | 0.224 | [ 2001.07:2001.48] 0.330 [ 2001.80:2002.87] 0.670 | [ 1957.50:1958.00] 0.068 [ 2000.42:2000.47] 0.003 [ 2000.98:2003.80] 0.927 [ 2004.01:2004.05] 0.002 | 1957:1957 | 1957:1958 |
| 8270 | 110.393 | 0.261 | [ 1997.03:1999.13] 0.869 [ 1999.48:1999.85] 0.131 | [ 1957.73:1958.15] 0.035 [ 1996.06:1996.64] 0.040 [ 1996.93:2000.07] 0.925 | 1957:1958 | 1957:1958 |
| 8271 | 109.769 | 0.241 | [ 1998.22:1998.39] 0.087 [ 1998.83:2000.18] 0.797 [ 2000.45:2000.68] 0.116 | [ 1957.63:1958.19] 0.066 [ 1997.25:1997.31] 0.007 [ 1997.84:1997.90] 0.004 [ 1998.14:2001.04] 0.923 | 1957:1958 | 1957:1958 |
| 8286 | 110.516 | 0.249 | [ 1996.98:1998.99] 0.948 [ 1999.52:1999.83] 0.052 | [ 1957.74:1958.18] 0.034 [ 1996.05:2000.05] 0.966 | 1957:1958 | 1957:1958 |
| 8287 | 117.439 | 0.265 | [ 1987.19:1987.21] 0.009 [ 1987.91:1989.65] 0.991 | [ 1958.52:1959.08] 0.069 [ 1985.84:1985.90] 0.006 [ 1986.25:1986.31] 0.006 [ 1987.07:1987.34] 0.052 [ 1987.81:1989.91] 0.868 | 1958:1958 | 1958:1958 |
| 8288 | 122.37 | 0.265 | [ 1959.32:1959.45] 0.051 [ 1959.96:1961.57] 0.735 [ 1983.07:1983.11] 0.013 [ 1983.31:1983.38] 0.023 [ 1983.79:1983.99] 0.084 [ 1984.42:1984.75] 0.095 | [ 1959.15:1961.61] 0.665 [ 1982.85:1985.21] 0.328 [ 1985.50:1985.70] 0.006 | 1958:1958 | 1958:1958 |
| 8289 | 111.808 | 0.336 | [ 1994.07:1994.36] 0.094 [ 1994.76:1996.60] 0.906 | [ 1957.85:1958.47] 0.064 [ 1993.82:1996.94] 0.927 [ 1997.14:1997.23] 0.009 | 1958:1958 | 1958:1958 |
| 8290 | 111.702 | 0.251 | [ 1994.11:1994.31] 0.023 [ 1994.93:1996.86] 0.977 | [ 1957.83:1958.47] 0.071 [ 1993.83:1994.52] 0.098 [ 1994.73:1996.93] 0.819 [ 1997.13:1997.23] 0.011 | 1958:1958 | 1958:1958 |
| 8291 | 112.928 | 0.248 | [ 1993.11:1994.90] 1.000 | [ 1957.98:1958.60] 0.061 [ 1992.06:1992.37] 0.034 [ 1992.92:1995.08] 0.876 [ 1995.59:1995.81] 0.029 | 1958:1958 | 1958:1959 |
| 8292 | 117.439 | 0.265 | [ 1987.19:1987.21] 0.009 [ 1987.91:1989.65] 0.991 | [ 1958.52:1959.08] 0.069 [ 1985.84:1985.90] 0.006 [ 1986.25:1986.31] 0.006 [ 1987.07:1987.34] 0.052 [ 1987.81:1989.91] 0.868 | 1958:1958 | 1958:1959 |
| 8293 | 122.398 | 0.261 | [ 1959.32:1959.45] 0.051 [ 1959.91:1961.57] 0.753 [ 1983.06:1983.38] 0.044 [ 1983.79:1984.00] 0.088 [ 1984.44:1984.74] 0.065 | [ 1959.15:1961.61] 0.671 [ 1982.85:1985.21] 0.325 [ 1985.51:1985.70] 0.004 | 1958:1959 | 1958:1961 |
| 8294 | 123.631 | 0.252 | [ 1959.44:1960.07] 0.312 [ 1961.57:1961.68] 0.064 [ 1982.19:1983.80] 0.624 | [ 1959.34:1960.98] 0.377 [ 1961.54:1961.72] 0.055 [ 1982.14:1984.12] 0.561 [ 1984.90:1984.98] 0.007 | 1958:1961 | 1958:1961 |
| 8295 | 128.467 | 0.281 | [ 1961.91:1962.12] 0.215 [ 1979.36:1980.05] 0.544 [ 1980.43:1980.74] 0.241 | [ 1961.87:1962.21] 0.187 [ 1979.17:1980.82] 0.802 [ 1981.45:1981.53] 0.011 | 1961:1962 | 1958:1962 |
| 8296 | 122.54 | 0.276 | [ 1959.33:1959.47] 0.056 [ 1959.77:1961.58] 0.775 [ 1983.05:1983.39] 0.075 [ 1983.76:1983.98] 0.090 [ 1984.69:1984.70] 0.003 | [ 1959.18:1961.62] 0.673 [ 1982.52:1982.53] 0.001 [ 1982.83:1985.02] 0.327 | 1959:1962 | 1958:1962 |
| 8297 | 122.909 | 0.286 | [ 1959.37:1961.13] 0.662 [ 1961.51:1961.61] 0.055 [ 1982.96:1983.42] 0.194 [ 1983.67:1983.86] 0.089 | [ 1959.25:1961.65] 0.621 [ 1982.27:1982.28] 0.001 [ 1982.48:1982.56] 0.008 [ 1982.80:1984.17] 0.342 [ 1984.52:1985.01] 0.028 | 1959:1962 | 1959:1962 |
| 8298 | 123.583 | 0.291 | [ 1959.43:1960.15] 0.341 [ 1961.56:1961.67] 0.061 [ 1982.20:1982.24] 0.013 [ 1982.47:1982.57] 0.034 [ 1982.78:1983.80] 0.552 | [ 1959.34:1961.03] 0.391 [ 1961.54:1961.72] 0.054 [ 1982.14:1984.12] 0.547 [ 1984.90:1984.99] 0.008 | 1959:1962 | 1959:1962 |
| 8299 | 123.615 | 0.293 | [ 1959.43:1960.09] 0.318 [ 1961.56:1961.68] 0.065 [ 1982.19:1982.26] 0.024 [ 1982.47:1983.80] 0.594 | [ 1959.34:1961.02] 0.383 [ 1961.54:1961.72] 0.055 [ 1982.14:1984.12] 0.555 [ 1984.90:1984.99] 0.008 | 1959:1962 | 1959:1962 |
| 8325 | 122.48 | 0.3 | [ 1959.32:1959.46] 0.055 [ 1959.81:1961.58] 0.761 [ 1983.05:1983.39] 0.069 [ 1983.77:1984.00] 0.092 [ 1984.65:1984.72] 0.023 | [ 1959.17:1961.62] 0.666 [ 1982.84:1985.20] 0.332 [ 1985.52:1985.54] 0.001 | 1960:1962 | 1960:1962 |
| 8326 | 121.317 | 0.289 | [ 1959.14:1959.37] 0.080 [ 1983.86:1985.75] 0.920 | [ 1958.98:1959.42] 0.081 [ 1960.11:1961.51] 0.198 [ 1983.83:1986.18] 0.722 | 1960:1962 | 1960:1962 |
| 8327 | 121.754 | 0.292 | [ 1959.25:1959.39] 0.049 [ 1960.71:1961.52] 0.343 [ 1983.84:1984.89] 0.508 [ 1985.47:1985.71] 0.100 | [ 1959.05:1959.45] 0.067 [ 1959.85:1961.55] 0.376 [ 1983.00:1983.08] 0.006 [ 1983.31:1983.39] 0.007 [ 1983.79:1985.75] 0.543 [ 1986.10:1986.12] 0.001 | 1961:1962 | 1960:1962 |
| 8328 | 122.468 | 0.285 | [ 1959.32:1959.46] 0.055 [ 1959.83:1961.58] 0.763 [ 1983.05:1983.39] 0.065 [ 1983.77:1983.99] 0.090 [ 1984.65:1984.73] 0.027 | [ 1959.17:1961.61] 0.670 [ 1982.84:1985.20] 0.328 [ 1985.51:1985.70] 0.002 | 1961:1961 | 1961:1962 |
| 8329 | 124.88 | 0.286 | [ 1961.66:1961.79] 0.098 [ 1980.99:1981.35] 0.152 [ 1981.84:1982.82] 0.750 | [ 1959.49:1959.76] 0.009 [ 1961.61:1961.89] 0.091 [ 1980.11:1980.38] 0.026 [ 1980.68:1983.07] 0.835 [ 1983.38:1983.67] 0.030 [ 1983.99:1984.09] 0.005 [ 1984.91:1984.98] 0.005 | 1961:1962 | 1961:1962 |
| 8330 | 127.904 | 0.295 | [ 1961.88:1962.05] 0.158 [ 1979.87:1980.80] 0.842 | [ 1961.83:1962.16] 0.153 [ 1979.29:1980.85] 0.778 [ 1981.08:1981.14] 0.012 [ 1981.40:1981.97] 0.057 | 1961:1962 | 1961:1962 |
| 8332 | 132.355 | 0.347 | [ 1962.26:1962.37] 0.060 [ 1977.11:1977.12] 0.005 [ 1977.78:1978.97] 0.935 | [ 1962.15:1962.52] 0.105 [ 1976.17:1976.25] 0.012 [ 1976.77:1979.00] 0.883 | 1962:1962 | 1962:1962 |
| 8333 | 133.791 | 0.318 | [ 1976.25:1976.29] 0.022 [ 1976.74:1977.85] 0.849 [ 1978.39:1978.54] 0.129 | [ 1962.25:1962.58] 0.080 [ 1976.14:1978.88] 0.920 | 1962:1962 | 1962:1962 |
| 8334 | 145.389 | 0.351 | [ 1962.90:1963.03] 0.206 [ 1971.51:1971.54] 0.034 [ 1972.52:1972.54] 0.016 [ 1972.96:1973.87] 0.743 | [ 1962.84:1963.07] 0.172 [ 1970.10:1970.13] 0.005 [ 1971.10:1971.28] 0.022 [ 1971.49:1971.57] 0.040 [ 1972.21:1973.88] 0.759 [ 1974.61:1974.62] 0.001 | 1962:1963 | 1962:1963 |
| 8335 | 150.625 | 0.371 | [ 1963.15:1963.21] 0.070 [ 1969.69:1969.70] 0.006 [ 1970.02:1970.21] 0.086 [ 1970.55:1971.06] 0.340 [ 1971.32:1972.17] 0.497 | [ 1963.11:1963.24] 0.068 [ 1968.89:1968.99] 0.024 [ 1969.67:1969.72] 0.016 [ 1970.00:1972.46] 0.887 [ 1972.77:1972.81] 0.004 | 1963:1963 | 1963:1963 |
| 8336 | 157.054 | 0.366 | [ 1963.28:1963.30] 0.015 [ 1968.18:1968.83] 0.495 [ 1969.03:1969.43] 0.436 [ 1969.78:1969.82] 0.032 [ 1970.34:1970.37] 0.022 | [ 1963.25:1963.32] 0.023 [ 1967.00:1967.07] 0.011 [ 1967.36:1967.38] 0.002 [ 1967.69:1967.72] 0.003 [ 1967.98:1969.92] 0.915 [ 1970.27:1970.40] 0.045 | 1963:1963 | 1963:1963 |
| 8337 | 160.137 | 0.367 | [ 1967.48:1968.16] 0.835 [ 1968.58:1968.68] 0.165 | [ 1963.29:1963.34] 0.027 [ 1966.97:1967.10] 0.048 [ 1967.32:1968.73] 0.925 | 1963:1963 | 1963:1963 |
| 8338 | 163.39 | 0.359 | [ 1963.32:1963.35] 0.032 [ 1966.76:1967.66] 0.968 | [ 1963.30:1963.39] 0.040 [ 1965.68:1965.72] 0.005 [ 1965.92:1967.91] 0.955 | 1963:1963 | 1963:1963 |
| 8339 | 165.768 | 0.37 | [ 1963.34:1963.37] 0.026 [ 1965.97:1967.32] 0.967 [ 1967.61:1967.62] 0.006 | [ 1963.33:1963.40] 0.034 [ 1965.67:1967.63] 0.966 | 1963:1963 | 1963:1963 |
| 8340 | 166.576 | 0.378 | [ 1963.35:1963.37] 0.018 [ 1965.96:1966.96] 0.973 [ 1967.27:1967.28] 0.009 | [ 1963.33:1963.42] 0.040 [ 1965.66:1967.62] 0.960 | 1963:1963 | 1963:1963 |
| 8341 | 168.575 | 0.389 | [ 1963.36:1963.41] 0.056 [ 1965.91:1966.67] 0.944 | [ 1963.34:1963.45] 0.062 [ 1963.76:1963.89] 0.020 [ 1964.16:1964.23] 0.013 [ 1965.63:1967.03] 0.901 [ 1967.33:1967.38] 0.005 | 1963:1963 | 1963:1963 |
| 8342 | 169.941 | 0.379 | [ 1963.37:1963.43] 0.080 [ 1964.17:1964.20] 0.030 [ 1965.63:1966.64] 0.890 | [ 1963.35:1963.46] 0.078 [ 1963.75:1963.90] 0.033 [ 1964.14:1964.26] 0.052 [ 1965.17:1966.68] 0.832 [ 1966.93:1966.97] 0.005 | 1963:1963 | 1963:1963 |
| 8343 | 169.964 | 0.403 | [ 1963.37:1963.43] 0.079 [ 1964.17:1964.20] 0.030 [ 1965.63:1966.64] 0.891 | [ 1963.35:1963.46] 0.078 [ 1963.75:1963.90] 0.033 [ 1964.14:1964.26] 0.053 [ 1965.17:1966.68] 0.832 [ 1966.93:1966.96] 0.003 | 1963:1963 | 1963:1963 |
| 8344 | 178.184 | 0.398 | [ 1963.45:1963.48] 0.025 [ 1963.98:1964.39] 0.322 [ 1964.74:1965.06] 0.476 [ 1965.34:1965.55] 0.177 | [ 1963.43:1964.40] 0.369 [ 1964.65:1965.58] 0.614 [ 1965.81:1965.89] 0.017 | 1963:1963 | 1963:1963 |
| 8345 | 185.98 | 0.413 | [ 1963.49:1963.55] 0.093 [ 1963.93:1963.95] 0.027 [ 1964.42:1964.69] 0.880 | [ 1963.48:1963.96] 0.312 [ 1964.42:1964.76] 0.688 | 1963:1964 | 1963:1964 |
| 8346 | 184.671 | 0.407 | [ 1963.48:1963.52] 0.059 [ 1963.92:1963.97] 0.096 [ 1964.41:1964.75] 0.844 | [ 1963.47:1963.99] 0.354 [ 1964.27:1964.78] 0.646 | 1963:1963 | 1963:1964 |
| 8347 | 187.382 | 0.404 | [ 1963.49:1963.72] 0.165 [ 1964.44:1964.68] 0.835 | [ 1963.48:1963.94] 0.354 [ 1964.43:1964.74] 0.646 | 1963:1963 | 1963:1964 |
| 8348 | 177.73 | 0.402 | [ 1963.45:1963.48] 0.023 [ 1963.99:1964.39] 0.279 [ 1964.80:1965.09] 0.512 [ 1965.33:1965.55] 0.186 | [ 1963.43:1963.50] 0.029 [ 1963.72:1964.40] 0.295 [ 1964.66:1965.59] 0.657 [ 1965.81:1965.89] 0.019 | 1963:1964 | 1963:1964 |
| 8405 | 180.554 | 0.445 | [ 1963.46:1963.50] 0.049 [ 1963.89:1964.04] 0.264 [ 1964.25:1964.42] 0.250 [ 1964.65:1964.91] 0.437 | [ 1963.45:1964.94] 0.919 [ 1965.29:1965.55] 0.079 [ 1965.86:1965.87] 0.002 | 1964:1964 | 1963:1964 |
| 8406 | 182.204 | 0.441 | [ 1963.47:1963.52] 0.069 [ 1963.89:1964.02] 0.274 [ 1964.27:1964.85] 0.657 | [ 1963.45:1964.91] 0.994 [ 1965.50:1965.53] 0.006 | 1964:1964 | 1963:1964 |
| 8407 | 181.506 | 0.441 | [ 1963.46:1963.51] 0.064 [ 1963.88:1964.03] 0.294 [ 1964.26:1964.88] 0.642 | [ 1963.45:1964.92] 0.971 [ 1965.41:1965.54] 0.029 | 1964:1964 | 1964:1964 |
| 8408 | 183.626 | 0.467 | [ 1963.47:1963.52] 0.070 [ 1963.90:1963.99] 0.188 [ 1964.40:1964.78] 0.742 | [ 1963.46:1964.01] 0.387 [ 1964.26:1964.86] 0.613 | 1964:1964 | 1964:1964 |
| 8409 | 183.014 | 0.43 | [ 1963.47:1963.52] 0.072 [ 1963.74:1964.00] 0.239 [ 1964.39:1964.81] 0.689 | [ 1963.46:1964.02] 0.403 [ 1964.25:1964.88] 0.597 | 1964:1964 | 1964:1964 |
| 8410 | 184.072 | 0.45 | [ 1963.48:1963.52] 0.061 [ 1963.91:1963.98] 0.145 [ 1964.40:1964.77] 0.795 | [ 1963.46:1964.01] 0.381 [ 1964.26:1964.82] 0.619 | 1964:1964 | 1964:1964 |
| 8411 | 185.825 | 0.425 | [ 1963.49:1963.54] 0.080 [ 1963.92:1963.95] 0.040 [ 1964.42:1964.69] 0.880 | [ 1963.48:1963.97] 0.315 [ 1964.42:1964.76] 0.685 | 1964:1964 | 1964:1964 |
| 8412 | 189.662 | 0.433 | [ 1963.50:1963.72] 0.575 [ 1964.53:1964.65] 0.425 | [ 1963.49:1963.92] 0.555 [ 1964.47:1964.72] 0.445 | 1964:1964 | 1964:1964 |
| 8413 | 177.774 | 0.44 | [ 1963.45:1963.48] 0.023 [ 1963.99:1964.39] 0.291 [ 1964.80:1965.09] 0.507 [ 1965.34:1965.55] 0.179 | [ 1963.43:1963.50] 0.030 [ 1963.72:1964.40] 0.298 [ 1964.66:1965.59] 0.654 [ 1965.81:1965.89] 0.019 | 1964:1965 | 1964:1965 |
| 8414 | 175.963 | 0.423 | [ 1964.03:1964.37] 0.136 [ 1964.88:1965.59] 0.864 | [ 1963.41:1963.49] 0.029 [ 1963.74:1964.38] 0.230 [ 1964.68:1965.91] 0.741 | 1964:1965 | 1964:1965 |
| 8415 | 170.25 | 0.408 | [ 1963.37:1963.44] 0.090 [ 1964.16:1964.21] 0.056 [ 1965.63:1966.63] 0.855 | [ 1963.35:1963.47] 0.081 [ 1963.75:1963.90] 0.035 [ 1964.13:1964.26] 0.065 [ 1964.86:1964.87] 0.001 [ 1965.17:1966.67] 0.817 | 1965:1965 | 1965:1966 |
| 8416 | 170.471 | 0.421 | [ 1963.37:1963.45] 0.098 [ 1964.15:1964.22] 0.080 [ 1965.62:1965.99] 0.432 [ 1966.20:1966.63] 0.390 | [ 1963.35:1963.47] 0.082 [ 1963.75:1963.90] 0.036 [ 1964.13:1964.26] 0.076 [ 1964.86:1964.87] 0.001 [ 1965.17:1966.66] 0.805 | 1965:1966 | 1965:1966 |
| 8417 | 170.183 | 0.37 | [ 1963.37:1963.44] 0.091 [ 1964.16:1964.21] 0.054 [ 1965.63:1966.63] 0.855 | [ 1963.35:1963.47] 0.082 [ 1963.75:1963.90] 0.034 [ 1964.13:1964.26] 0.063 [ 1964.86:1964.87] 0.001 [ 1965.17:1966.67] 0.820 | 1965:1966 | 1965:1966 |
| 8418 | 169.668 | 0.349 | [ 1963.36:1963.43] 0.085 [ 1964.19:1964.20] 0.009 [ 1965.64:1966.64] 0.906 | [ 1963.34:1963.46] 0.077 [ 1963.75:1963.90] 0.030 [ 1964.14:1964.26] 0.042 [ 1965.18:1966.68] 0.841 [ 1966.92:1966.99] 0.009 | 1965:1966 | 1965:1966 |
| 8419 | 170.9 | 0.371 | [ 1963.37:1963.45] 0.097 [ 1964.13:1964.23] 0.121 [ 1965.19:1965.23] 0.028 [ 1965.62:1965.98] 0.440 [ 1966.21:1966.62] 0.314 | [ 1963.35:1963.47] 0.081 [ 1963.75:1963.90] 0.039 [ 1964.12:1964.27] 0.100 [ 1964.86:1964.87] 0.001 [ 1965.16:1966.64] 0.779 | 1966:1966 | 1965:1966 |
| 8421 | 165.466 | 0.355 | [ 1963.34:1963.37] 0.027 [ 1965.99:1967.33] 0.965 [ 1967.61:1967.62] 0.009 | [ 1963.32:1963.40] 0.035 [ 1965.68:1967.64] 0.965 | 1966:1966 | 1966:1967 |
| 8422 | 164.367 | 0.361 | [ 1963.33:1963.36] 0.028 [ 1966.11:1966.12] 0.007 [ 1966.68:1967.64] 0.965 | [ 1963.31:1963.39] 0.035 [ 1965.68:1967.66] 0.965 | 1966:1967 | 1966:1967 |
| 8423 | 165.188 | 0.354 | [ 1963.33:1963.37] 0.033 [ 1966.05:1966.44] 0.181 [ 1966.65:1967.34] 0.757 [ 1967.60:1967.63] 0.029 | [ 1963.32:1963.40] 0.035 [ 1965.67:1967.64] 0.965 | 1966:1967 | 1966:1967 |
| 8424 | 164.491 | 0.367 | [ 1963.33:1963.36] 0.027 [ 1966.10:1966.13] 0.021 [ 1966.67:1967.36] 0.875 [ 1967.58:1967.64] 0.076 | [ 1963.31:1963.39] 0.035 [ 1965.68:1967.66] 0.965 | 1966:1967 | 1966:1967 |
| 8425 | 165.527 | 0.369 | [ 1963.34:1963.37] 0.027 [ 1965.98:1967.33] 0.965 [ 1967.61:1967.62] 0.008 | [ 1963.32:1963.40] 0.035 [ 1965.68:1967.63] 0.965 | 1966:1967 | 1966:1967 |
| 8461 | 164.329 | 0.36 | [ 1963.33:1963.36] 0.028 [ 1966.68:1967.64] 0.972 | [ 1963.31:1963.39] 0.035 [ 1965.68:1967.66] 0.965 | 1967:1967 | 1967:1967 |
| 8462 | 162.14 | 0.357 | [ 1963.31:1963.34] 0.038 [ 1966.97:1967.92] 0.954 [ 1968.61:1968.62] 0.008 | [ 1963.29:1963.37] 0.044 [ 1965.69:1965.70] 0.001 [ 1966.32:1966.46] 0.023 [ 1966.72:1968.10] 0.905 [ 1968.59:1968.66] 0.026 | 1967:1967 | 1967:1967 |
| 8463 | 159.993 | 0.337 | [ 1967.48:1968.16] 0.818 [ 1968.58:1968.69] 0.182 | [ 1963.28:1963.34] 0.028 [ 1966.97:1967.10] 0.046 [ 1967.32:1968.73] 0.924 [ 1969.36:1969.37] 0.001 | 1967:1967 | 1967:1967 |
| 8464 | 160.487 | 0.372 | [ 1963.31:1963.32] 0.010 [ 1967.46:1968.14] 0.851 [ 1968.58:1968.67] 0.139 | [ 1963.29:1963.35] 0.030 [ 1966.97:1967.10] 0.054 [ 1967.31:1968.21] 0.790 [ 1968.56:1968.71] 0.125 | 1967:1967 | 1967:1968 |
| 8465 | 160.505 | 0.357 | [ 1963.31:1963.32] 0.010 [ 1967.46:1968.14] 0.852 [ 1968.58:1968.67] 0.138 | [ 1963.29:1963.35] 0.030 [ 1966.97:1967.10] 0.054 [ 1967.31:1968.21] 0.791 [ 1968.56:1968.71] 0.124 | 1967:1968 | 1967:1968 |
| 8466 | 160.425 | 0.366 | [ 1963.31:1963.32] 0.010 [ 1967.46:1968.14] 0.848 [ 1968.58:1968.67] 0.142 | [ 1963.29:1963.35] 0.030 [ 1966.97:1967.10] 0.053 [ 1967.32:1968.21] 0.786 [ 1968.56:1968.72] 0.131 | 1967:1968 | 1967:1968 |
| 8467 | 159.806 | 0.348 | [ 1967.49:1968.18] 0.799 [ 1968.57:1968.69] 0.201 | [ 1963.28:1963.34] 0.028 [ 1966.97:1967.10] 0.044 [ 1967.32:1968.75] 0.924 [ 1969.36:1969.38] 0.004 | 1967:1968 | 1967:1968 |
| 8468 | 157.337 | 0.367 | [ 1963.28:1963.30] 0.015 [ 1968.02:1968.81] 0.571 [ 1969.04:1969.42] 0.406 [ 1970.36:1970.37] 0.007 | [ 1963.26:1963.32] 0.023 [ 1967.00:1967.07] 0.013 [ 1967.36:1967.41] 0.006 [ 1967.69:1969.90] 0.919 [ 1970.28:1970.40] 0.038 | 1967:1968 | 1967:1968 |
| 8469 | 156.867 | 0.36 | [ 1963.28:1963.29] 0.007 [ 1968.19:1968.83] 0.464 [ 1969.03:1969.44] 0.438 [ 1969.78:1969.85] 0.061 [ 1970.34:1970.38] 0.031 | [ 1963.25:1963.32] 0.023 [ 1967.01:1967.06] 0.008 [ 1967.98:1969.93] 0.918 [ 1970.27:1970.41] 0.051 | 1967:1968 | 1967:1968 |
| 8470 | 156.274 | 0.339 | [ 1968.23:1968.54] 0.245 [ 1968.75:1969.57] 0.586 [ 1969.77:1969.90] 0.143 [ 1970.34:1970.37] 0.026 | [ 1963.25:1963.31] 0.021 [ 1967.02:1967.05] 0.004 [ 1968.00:1969.95] 0.909 [ 1970.26:1970.41] 0.066 | 1968:1968 | 1968:1969 |
| 8471 | 156.411 | 0.358 | [ 1968.22:1969.54] 0.833 [ 1969.77:1969.89] 0.126 [ 1970.33:1970.38] 0.042 | [ 1963.25:1963.31] 0.021 [ 1967.02:1967.05] 0.004 [ 1968.00:1969.95] 0.912 [ 1970.26:1970.41] 0.063 | 1968:1969 | 1968:1969 |
| 8472 | 157 | 0.342 | [ 1963.28:1963.30] 0.014 [ 1968.18:1968.83] 0.492 [ 1969.03:1969.43] 0.437 [ 1969.78:1969.83] 0.041 [ 1970.35:1970.37] 0.015 | [ 1963.25:1963.32] 0.023 [ 1967.00:1967.07] 0.011 [ 1967.36:1967.37] 0.001 [ 1967.69:1967.70] 0.001 [ 1967.98:1969.92] 0.918 [ 1970.27:1970.40] 0.046 | 1968:1969 | 1968:1969 |
| 8473 | 157.038 | 0.356 | [ 1963.28:1963.30] 0.015 [ 1968.18:1968.83] 0.498 [ 1969.03:1969.43] 0.440 [ 1969.78:1969.82] 0.033 [ 1970.35:1970.37] 0.015 | [ 1963.25:1963.32] 0.023 [ 1967.00:1967.07] 0.011 [ 1967.36:1967.38] 0.002 [ 1967.69:1967.71] 0.002 [ 1967.98:1969.92] 0.916 [ 1970.27:1970.40] 0.045 | 1968:1969 | 1968:1969 |
| 8474 | 156.843 | 0.485 | [ 1963.28:1963.30] 0.014 [ 1968.19:1969.44] 0.879 [ 1969.78:1969.86] 0.070 [ 1970.33:1970.38] 0.037 | [ 1963.25:1963.32] 0.023 [ 1967.01:1967.06] 0.008 [ 1967.98:1969.95] 0.917 [ 1970.27:1970.41] 0.052 | 1968:1969 | 1968:1969 |
| 8475 | 154.073 | 0.341 | [ 1963.23:1963.28] 0.049 [ 1968.41:1968.49] 0.107 [ 1968.83:1969.03] 0.125 [ 1969.25:1969.99] 0.484 [ 1970.23:1970.44] 0.188 [ 1970.76:1970.80] 0.046 | [ 1963.21:1963.30] 0.043 [ 1967.03:1967.04] 0.001 [ 1968.26:1968.53] 0.096 [ 1968.78:1970.83] 0.846 [ 1971.98:1972.06] 0.014 | 1969:1969 | 1969:1969 |
| 8476 | 154.653 | 0.352 | [ 1963.25:1963.27] 0.017 [ 1968.41:1968.50] 0.119 [ 1968.82:1969.03] 0.105 [ 1969.24:1969.98] 0.619 [ 1970.25:1970.42] 0.140 | [ 1963.22:1963.30] 0.033 [ 1968.24:1968.54] 0.125 [ 1968.76:1970.00] 0.681 [ 1970.22:1970.54] 0.143 [ 1970.75:1970.81] 0.016 [ 1972.02:1972.04] 0.002 | 1969:1969 | 1969:1970 |
| 8477 | 152.555 | 0.486 | [ 1963.20:1963.26] 0.063 [ 1968.88:1969.00] 0.099 [ 1969.64:1969.75] 0.141 [ 1969.97:1970.85] 0.585 [ 1971.36:1971.40] 0.026 [ 1971.68:1971.72] 0.028 [ 1971.99:1972.07] 0.058 | [ 1963.19:1963.29] 0.055 [ 1968.42:1968.48] 0.013 [ 1968.84:1969.03] 0.088 [ 1969.26:1969.29] 0.004 [ 1969.62:1971.07] 0.639 [ 1971.32:1972.20] 0.201 | 1969:1970 | 1969:1970 |
| 8478 | 151.381 | 0.325 | [ 1963.18:1963.23] 0.056 [ 1969.67:1969.72] 0.049 [ 1970.00:1970.98] 0.504 [ 1971.34:1972.11] 0.391 | [ 1963.15:1963.26] 0.056 [ 1968.88:1969.00] 0.041 [ 1969.65:1969.74] 0.045 [ 1969.98:1972.23] 0.858 | 1969:1970 | 1969:1970 |
| 8479 | 152.908 | 0.324 | [ 1963.21:1963.27] 0.068 [ 1968.44:1968.46] 0.017 [ 1968.87:1969.01] 0.112 [ 1969.63:1969.75] 0.167 [ 1969.96:1970.84] 0.614 [ 1972.02:1972.05] 0.022 | [ 1963.20:1963.29] 0.056 [ 1967.03:1967.04] 0.001 [ 1968.41:1968.49] 0.025 [ 1968.82:1969.03] 0.106 [ 1969.25:1970.94] 0.676 [ 1971.33:1971.45] 0.032 [ 1971.65:1972.10] 0.105 | 1969:1970 | 1969:1970 |
| 8480 | 153.942 | 0.324 | [ 1963.23:1963.28] 0.052 [ 1968.42:1968.49] 0.099 [ 1968.84:1969.03] 0.137 [ 1969.26:1970.00] 0.418 [ 1970.23:1970.53] 0.221 [ 1970.75:1970.81] 0.072 | [ 1963.21:1963.30] 0.046 [ 1967.03:1967.04] 0.001 [ 1968.26:1968.53] 0.087 [ 1968.78:1970.83] 0.847 [ 1971.97:1972.07] 0.019 | 1970:1970 | 1969:1970 |
| 8481 | 153.325 | 0.334 | [ 1963.21:1963.27] 0.064 [ 1968.43:1968.47] 0.046 [ 1968.86:1969.02] 0.138 [ 1969.27:1969.28] 0.007 [ 1969.62:1970.01] 0.265 [ 1970.21:1970.83] 0.481 | [ 1963.20:1963.29] 0.054 [ 1967.03:1967.04] 0.001 [ 1968.41:1968.51] 0.048 [ 1968.80:1970.85] 0.821 [ 1971.34:1971.45] 0.017 [ 1971.67:1972.09] 0.059 | 1970:1970 | 1969:1970 |
| 8482 | 152.813 | 0.344 | [ 1963.21:1963.26] 0.060 [ 1968.44:1968.45] 0.007 [ 1968.87:1969.01] 0.123 [ 1969.63:1969.75] 0.163 [ 1969.96:1970.84] 0.610 [ 1972.01:1972.06] 0.036 | [ 1963.20:1963.29] 0.056 [ 1967.03:1967.04] 0.001 [ 1968.42:1968.49] 0.021 [ 1968.82:1969.03] 0.101 [ 1969.25:1971.00] 0.667 [ 1971.32:1972.10] 0.154 | 1970:1970 | 1969:1970 |
| 8483 | 152.723 | 0.351 | [ 1963.21:1963.26] 0.060 [ 1968.44:1968.45] 0.006 [ 1968.88:1969.01] 0.100 [ 1969.63:1969.75] 0.160 [ 1969.97:1970.85] 0.623 [ 1971.69:1971.70] 0.006 [ 1972.00:1972.06] 0.044 | [ 1963.20:1963.29] 0.056 [ 1968.42:1968.49] 0.018 [ 1968.83:1969.03] 0.097 [ 1969.26:1971.04] 0.662 [ 1971.32:1972.20] 0.168 | 1970:1970 | 1970:1970 |
| 8484 | 150.824 | 0.327 | [ 1963.16:1963.22] 0.069 [ 1969.68:1969.70] 0.013 [ 1970.01:1970.21] 0.088 [ 1970.54:1971.05] 0.350 [ 1971.33:1972.15] 0.480 | [ 1963.12:1963.25] 0.066 [ 1968.89:1969.00] 0.029 [ 1969.66:1969.73] 0.023 [ 1970.00:1972.45] 0.883 | 1970:1970 | 1970:1971 |
| 8485 | 151.146 | 0.349 | [ 1963.17:1963.23] 0.065 [ 1969.67:1969.71] 0.033 [ 1970.00:1970.23] 0.110 [ 1970.44:1971.03] 0.363 [ 1971.33:1972.11] 0.429 | [ 1963.14:1963.26] 0.060 [ 1968.88:1969.00] 0.036 [ 1969.65:1969.73] 0.033 [ 1969.99:1972.24] 0.871 | 1970:1970 | 1970:1971 |
| 8486 | 151.766 | 0.329 | [ 1963.19:1963.24] 0.055 [ 1968.91:1968.98] 0.035 [ 1969.66:1969.73] 0.080 [ 1969.99:1970.90] 0.524 [ 1971.35:1971.45] 0.101 [ 1971.65:1972.09] 0.204 | [ 1963.16:1963.27] 0.053 [ 1968.87:1969.01] 0.054 [ 1969.64:1969.75] 0.065 [ 1969.98:1971.10] 0.493 [ 1971.31:1972.22] 0.334 | 1970:1971 | 1970:1971 |
| 8487 | 152.306 | 0.333 | [ 1963.20:1963.25] 0.058 [ 1968.89:1968.99] 0.067 [ 1969.65:1969.74] 0.122 [ 1969.98:1970.86] 0.594 [ 1971.36:1971.45] 0.070 [ 1971.68:1971.72] 0.033 [ 1972.00:1972.07] 0.056 | [ 1963.18:1963.28] 0.054 [ 1968.43:1968.46] 0.005 [ 1968.86:1969.02] 0.076 [ 1969.27:1969.28] 0.001 [ 1969.63:1971.07] 0.628 [ 1971.32:1972.20] 0.237 | 1970:1971 | 1970:1971 |

Table S7. Oxcal modelling code

| IntCa13 | Pearson 2018 |
| --- | --- |
| **Ring count accurate**  Plot()  {  D_Sequence()  {  R_Date("Hd-23599-24426 1-13", 3383, 11);  Gap(18.5);  R_Date("Hd-23587 14-37", 3372, 12);  Gap(23);  R_Date("Hd-23589 38-59", 3349, 12);  Gap(17.5);  R_Date("Hd-23588-24402 60-72", 3331, 10);  };  }; | **Ring count accurate**  Plot()  {  Curve("Pearson2018", "Pearson2018.14c");  D_Sequence()  {  R_Date("Hd-23599-24426 1-13", 3383, 11);  Gap(18.5);  R_Date("Hd-23587 14-37", 3372, 12);  Gap(23);  R_Date("Hd-23589 38-59", 3349, 12);  Gap(17.5);  R_Date("Hd-23588-24402 60-72", 3331, 10);  };  }; |
| **Ring count is increased by 25%, and gap uncertainty set to 25% of section count**  Plot()  {  V_Sequence()  {  R_Date("Hd-23599-24426 1-13", 3383, 11);  Gap(23,6);  R_Date("Hd-23587 14-37", 3372, 12);  Gap(29,7);  R_Date("Hd-23589 38-59", 3349, 12);  Gap(22,5);  R_Date("Hd-23588-24402 60-72", 3331, 10);  };  }; | **Ring count is increased by 25%, and gap uncertainty set to 25% of section count**  Plot()  {  Curve("Pearson2018", "Pearson2018.14c");  V_Sequence()  {  R_Date("Hd-23599-24426 1-13", 3383, 11);  Gap(23,6);  R_Date("Hd-23587 14-37", 3372, 12);  Gap(29,7);  R_Date("Hd-23589 38-59", 3349, 12);  Gap(22,5);  R_Date("Hd-23588-24402 60-72", 3331, 10);  };  }; |
| **Considering only sequence**  Plot()  {  Sequence()  {  Boundary("S");  R_Date("innermost section",3383,11);  R_Date("second section",3372,12);  R_Date("third section",3349,12);  R_Date("outermost section",3331,10);  Boundary("E");  };  }; | **Considering only sequence**  Plot()  {  Curve("Pearson2018", "Pearson2018.14c");  Sequence()  {  Boundary("S");  R_Date("innermost section",3383,11);  R_Date("second section",3372,12);  R_Date("third section",3349,12);  R_Date("outermost section",3331,10);  Boundary("E");  };  }; |
| **Accounting for extra 20 rings in last ring**  Plot()  {  D_Sequence()  {  R_Date("Hd-23599-24426 1-13", 3383, 11);  Gap(18.5);  R_Date("Hd-23587 14-37", 3372, 12);  Gap(23);  R_Date("Hd-23589 38-59", 3349, 12);  Gap(32);  R_Date("Hd-23588-24402 60-72", 3331, 10);  };  }; | **Accounting for extra 20 rings in last ring**  Plot()  {  Curve("Pearson2018","Pearson2018.14c");  D_Sequence()  {  R_Date("Hd-23599-24426 1-13", 3383, 11);  Gap(18.5);  R_Date("Hd-23587 14-37", 3372, 12);  Gap(23);  R_Date("Hd-23589 38-59", 3349, 12);  Gap(32);  R_Date("Hd-23588-24402 60-72", 3331, 10);  };  }; |
